# Are Alkynyl Spacers in Ancillary Ligands in Heteroleptic Bis(diimine)copper(I) Dyes Beneficial for Dye Performance in Dye-Sensitized Solar Cells?

**DOI:** 10.3390/molecules25071528

**Published:** 2020-03-27

**Authors:** Guglielmo Risi, Mariia Becker, Catherine E. Housecroft, Edwin C. Constable

**Affiliations:** Department of Chemistry, University of Basel, BPR 1096, Mattenstrasse 24a, CH-4058 Basel, Switzerland; guglielmo.risi@unibas.ch (G.R.); mariia.karpacheva@unibas.ch (M.B.); catherine.housecroft@unibas.ch (C.E.H.)

**Keywords:** copper, dye-sensitized solar cell, 2,2′-bipyridine, alkynyl group, phosphonic acid anchor

## Abstract

The syntheses of 4,4′-bis(4-dimethylaminophenyl)-6,6′-dimethyl-2,2′-bipyridine (**1**), 4,4′-bis(4-dimethylaminophenylethynyl)-6,6′-dimethyl-2,2′-bipyridine (**2**), 4,4′-bis(4-diphenylaminophenyl)-6,6′-dimethyl-2,2′-bipyridine (**3**), and 4,4′-bis(4-diphenylaminophenylethynyl)-6,6′-dimethyl-2,2′-bipyridine (**4**) are reported along with the preparations and characterisations of their homoleptic copper(I) complexes [CuL_2_][PF_6_] (L = **1**–**4**). The solution absorption spectra of the complexes exhibit ligand-centred absorptions in addition to absorptions in the visible region assigned to a combination of intra-ligand and metal-to-ligand charge-transfer. Heteroleptic [Cu(**5**)(L_ancillary_)]^+^ dyes in which **5** is the anchoring ligand ((6,6′-dimethyl-[2,2′-bipyridine]-4,4′-diyl)bis(4,1-phenylene))bis(phosphonic acid) and L_ancillary_ = **1**–**4** have been assembled on fluorine-doped tin oxide (FTO)-TiO_2_ electrodes in dye-sensitized solar cells (DSCs). Performance parameters and external quantum efficiency (EQE) spectra of the DSCs (four fully-masked cells for each dye) reveal that the best performing dyes are [Cu(**5**)(**1**)]^+^ and [Cu(**5**)(**3**)]^+^. The alkynyl spacers are not beneficial, leading to a decrease in the short-circuit current density (*J*_SC_), confirmed by lower values of EQE_max_. Addition of a co-absorbent (*n*-decylphosphonic acid) to [Cu(**5**)(**1**)]^+^ lead to no significant enhancement of performance for DSCs sensitized with [Cu(**5**)(**1**)]^+^. Electrochemical impedance spectroscopy (EIS) has been used to investigate the interfaces in DSCs; the analysis shows that more favourable electron injection into TiO_2_ is observed for sensitizers without the alkynyl spacer and confirms higher *J*_SC_ values for [Cu(**5**)(**1**)]^+^.

## 1. Introduction

**Scheme 1:**
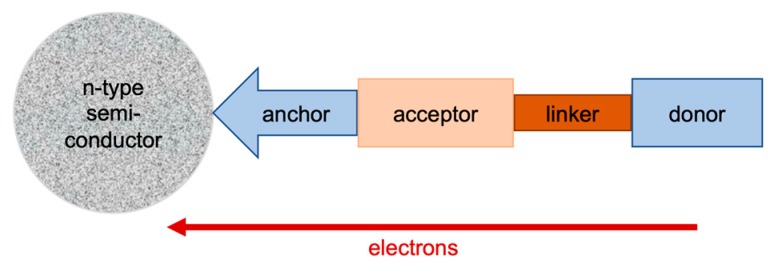
Schematic representation of a donor-acceptor (‘push-pull’) dye. In a heteroleptic bis(diimine)copper(I) dye, the anchoring ligand is the anchor/acceptor domain, the copper(I) centre is the linker, and the ancillary ligand is the donor.

Future energy strategies will rely increasingly upon sustainable methods of energy generation in order to conform to the United Nations Sustainable Development Goals. Emerging energy economies identify solar-to-electricity conversion as a key technology. Currently, the most widely deployed technologies are based upon semiconductor photovoltaics, but a promising methodology is the so-called dye-sensitized solar cell (DSC). DSCs [1] convert solar to electrical energy using a wide-bandgap semiconductor such as nanoparticulate TiO_2_ functionalized with a material that absorbs visible light [2,3,4,5]. The latter comprises the working electrode in an n-type DSC. Apart from the semiconductor and the dye, the other crucial components of the device are the electrolyte (incorporating a redox couple to facilitate electron and hole transport and to regenerate the ground-state of the dye after excitation), and the counter electrode (typically conductive glass coated with a thin layer of platinum to catalyse the regeneration of the reduced form of the redox couple). Some critical advances with DSCs using aqueous electrolytes have been made [6], but on the whole, electrolytes in the devices are based on organic solvents or polymers and the most popular redox shuttle in the electrolyte remains I_3_^−^/I^−^ [7]. Using metal-free (i.e., organic), ruthenium(II) or zinc(II) porphyrin sensitizers, it has been possible to achieve photoconversion efficiencies (PCE, *η*) of up to ca. 14% [8,9,10,11,12,13,14]. The parameters that characterize a DSC and contribute to the PCE are the open-circuit voltage (*V*_OC_) and the short-circuit current density (*J*_SC_). However, it is difficult to predict how a change in the molecular structure of a dye will influence *J*_SC_ and *V*_OC_. Theoretical approaches providing insight into these parameters are critical, for example, Quantum Theory of Atoms in Molecules (QTAIM) has been used to relate *J*_SC_ and *V*_OC_ to the electronic properties of organic dyes and to provide strategies for better organic dye design [15]. Significant efforts have also been made in fabricating alternative working electrodes, for example, by introducing graphene between the photoanode and dye, which leads to an increase in the rate of electron injection into the semiconductor [16]. Dye design typically centres on fulfilling ‘push-pull’ or donor-π-acceptor (D-π-A) characteristics (Scheme 1), but in addition, structural design should aim to suppress recombination pathways. A relevant example is the design of T-shaped donor-acceptor dyes incorporating phenoxazine/phenothiazine–triphenylamine donor domains [17].

While ruthenium dyes are still regarded as state-of-the-art, the abundance of ruthenium in the Earth’s crust is only about 0.001 ppm [18], making explorations of sensitizers incorporating Earth-abundant first-row metals, such as copper [19,20,21,22] and iron [23,24,25,26] extremely attractive. Significant progress has been made in the last few years in improving the PCEs of DSCs sensitized with copper(I) dyes. Although the best values of *η* are below 5% [27,28,29], systematic efforts in modifying the sensitizer, redox couple and other electrolyte components, and the use of co-sensitizers have allowed us [28,29,30,31] and others [27,32] to show that there is a viable future for copper(I) sensitizers.

**Scheme 2:**
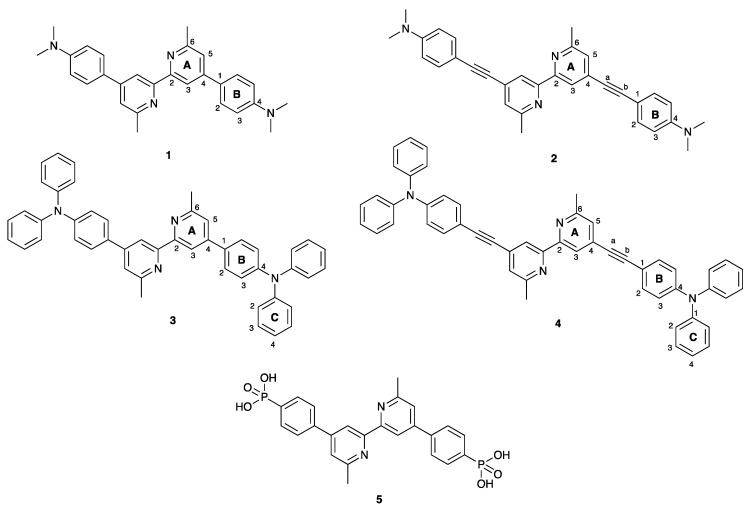
Structures of compounds **1**–**5**. Atom labelling for NMR spectroscopic assignments are given for **1**–**4**.

A key factor in performance enhancement of copper(I)-based DSCs is systematic modification of the structures of ‘push-pull’ dyes (Scheme 1). The ‘push-pull’ characteristics are readily tuned in a heteroleptic copper(I) complex (in which the Cu atom is the linker) through structural modification of the anchoring and ancillary ligands, such that in a charge-separated state, the hole is located on the ancillary ligand and the electron on the anchoring ligand. Donor-π-acceptor (D-π-A) dyes fulfil the ‘push-pull’ characteristics and introducing alkynyl units to optimize electronic communication and conjugation has been found to be beneficial in some families of dyes [33]. The +I nature of alkynyl substituents [34] increases the electron density on the metal centre and enhance the population of the metal-to-ligand charge transfer (MLCT) state [35]. Within the context of bis(diimine)copper(I) complexes, Castellano and co-workers demonstrated that the incorporation of phenylethynyl groups into the 4,7-positions of 2,3,7,8-tetraalkylsubstituted-1,10-phenanthrolines resulted in a significant red-shift in the MLCT absorptions and shifts to more positive potentials for both the copper(I) oxidation and ligand-based reduction processes compared to the parent bis(2,3,7,8-tetramethyl-1,10-phenanthroline)copper(I) complex. Most importantly, these beneficial effects were gained without loss in the long excited-state lifetimes, which result from the steric crowding imposed by the 2,3,7,8-tetraalkyl groups [36]. 2,2′-Bipyridine ligands bearing phenylethynyl or 4-substituted-phenylethynyl substituents in the 4,4′- or 5,5’-positions have been the focus of some attention, see for example [37,38,39,40], although, to the best of our knowledge, the use of related bis(diimine)copper(I) complexes in DSCs has not been explored. We now report the preparation and characterization of ligands **1**–**4** (Scheme 2), their homoleptic [CuL_2_][PF_6_] complexes, and the application of these compounds for the in situ assembly of heteroleptic copper(I) dyes on FTO/TiO_2_ photoanodes (FTO = fluorine-doped tin oxide) in DSCs. Ligands **1**–**4** contain peripheral electron-donating NR_2_ groups on the ancillary ligands, which are also known to be beneficial in stabilizing the hole remote from the semiconductor surface [21].

## 2. Results and Discussion

### 2.1. Ligand Syntheses and Characterization

**Scheme 3:**
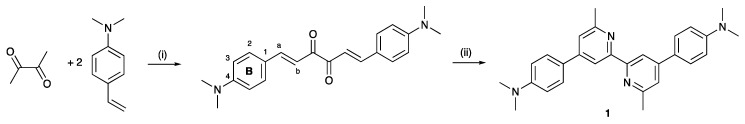
Reaction scheme for the preparation of **1**. Conditions: (i) piperidine, MeOH, reflux overnight; (ii) 1-(2-oxopropyl)pyridin-1-ium chloride, NH_4_OAc in EtOH, reflux overnight. Atom labelling for NMR spectroscopic assignments of the intermediate are given; see also Scheme 2.

**Scheme 4:**
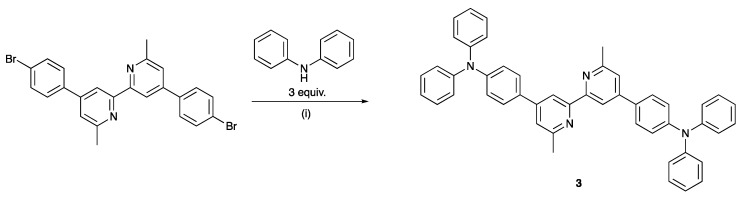
Reaction scheme for the preparation of **3**. Conditions: (i) Pd(*^t^*Bu_3_P)_2_, NaO*^t^*Bu, toluene, 100 °C overnight.

**Scheme 5:**
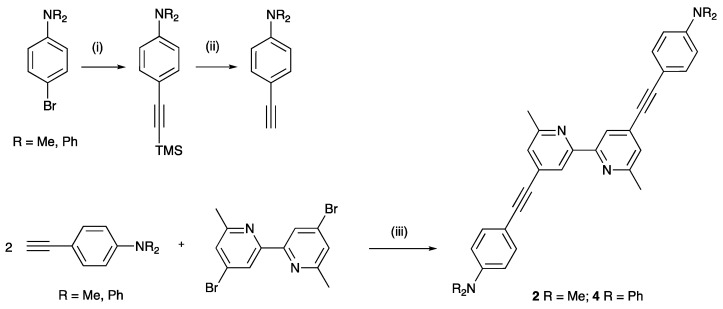
Reaction scheme for the preparations of **2** (R = Me) and **4** (R = Ph). Conditions: (i) Pd(dppf)Cl_2_, CuI, PPh_3_, Me_3_SiC≡CH in Et_3_N, 80 °C overnight; (ii) K_2_CO_3_ in MeOH, room temperature, 2 h; (iii) Pd(dppf)Cl_2_, CuI, PPh_3_ in Et_3_N, 80 °C overnight. (dppf = 1,1′-bis(diphenylphosphano)ferrocene).

Compound **1** was prepared using a Kröhnke approach [41] as shown in Scheme 3. For the diphenylamino-derivative **3**, the most convenient route was a palladium-catalysed coupling of 4,4′-bis(4-bromophenyl)-6,6′-dimethyl-2,2′-bipyridine and Ph_2_NH in the presence of base (Scheme 4) [42,43,44]. Scheme 5 summarizes the syntheses of ligands **2** and **4**. The high-resolution mass spectra of compounds **1**, **2**, **3** and **4** exhibited base peaks at *m*/*z* 423.2544, 471.2548, 671.3166 and 719.3162 corresponding to the [M + H]^+^ ions. The solid-state IR spectra of the ligands are shown in Figures S1–S4 (see Supporting Information). The presence of the alkynyl spacer gives rise to an absorption at 2198 cm^−1^ in **2** and 2207 cm^−1^ in **4**, the stretching mode being IR active due to the asymmetrical substitution.

The ^1^H and ^13^C{^1^H} NMR spectra of ligands **1**–**4** were assigned using two-dimensional (2D) methods. Figures S5–S8 (see Supporting Information) display the ^1^H NMR, NOESY, HMQC and HMBC spectra of compound **1**, and atom labelling is given in Scheme 2. Protons H^B2^ and H^B3^ are distinguished by the NOESY cross-peaks between signals for H^B2^ and H^A3^/H^A5^ and between H^B3^ and H^NMe^ (Figure S6). The NOESY cross-peak between the signals for H^A5^ and H^A6-Me^ (Figure S6) distinguished H^A5^ from H^A3^. The resonances for H^A5^ from H^A3^ were assigned in a similar manner in compounds **2**–**4**. In compounds **2** and **4**, the alkyne unit was characterized by ^13^C NMR resonances at *δ* 86.2 and 95.3 ppm in **2** and *δ* 86.**2** and 94.0 ppm in **4**; the signals were assigned from the HMBC cross-peaks to H^A5^ and H^B2^, respectively. NMR spectra for **2**–**4** are shown in Figures S9–S16 in the Supporting Information.

**Figure 1:**
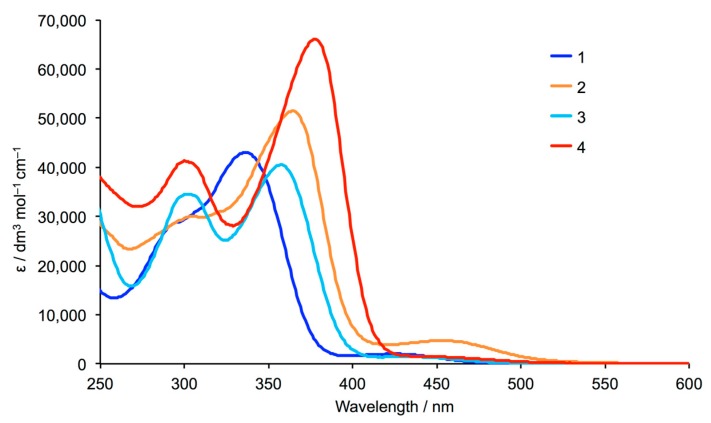
Solution absorption spectra of **1**–**4** (CHCl_3_, concentrations 2.1–2.6 × 10^−5^ mol dm^−3^).

The solution absorption spectra of compounds **1** and **2** (Figure 1) exhibit intense high-energy absorptions assigned to π*←π transitions [45]. Absorptions on the edge of the visible region are assigned to intra-ligand charge-transfer (ILCT) arising from transfer of charge from the electron-donating Me_2_N groups to the electron-poor bipyridine [21]. Introduction of NPh_2_ in place of NMe_2_ groups red-shifts the absorption spectra with a concomitant increase in values of the extinction coefficient (Figure 1). A comparison of the spectra of **2** with **1**, and of **4** with **3** shows the effect of extending the π-conjugation as the alkynyl unit is introduced, extending the absorption spectra towards longer wavelengths.

### 2.2. Syntheses and Characterization of Homoleptic Copper(I) Complexes

**Figure 2:**
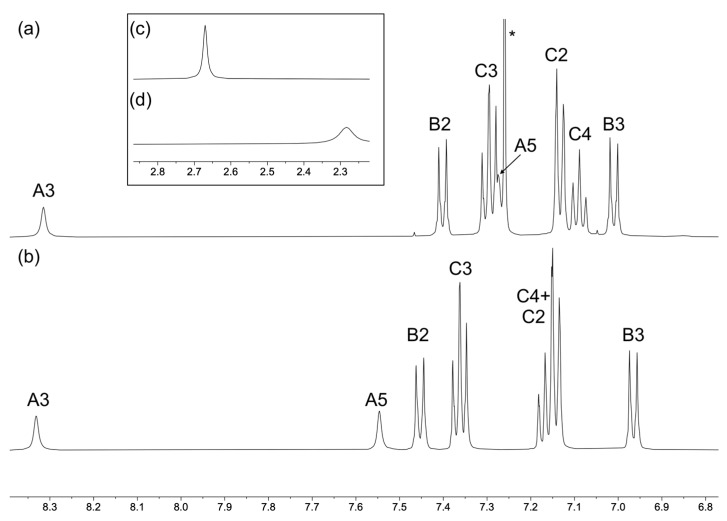
Comparison of the aromatic regions of the ^1^H NMR spectra (500 MHz, 298 K) of (**a**) ligand **4** in CDCl_3_ and (**b**) [Cu(**4**)_2_][PF_6_] in CD_3_CN. The alkyl regions are shown in (**c**) **4** and (**d**) [Cu(**4**)_2_][PF_6_]. See Scheme 2 for atom labelling. Scale: *δ/*ppm. * = residual CHCl_3_.

The [CuL_2_][PF_6_] compounds with L = **1**, **2**, **3** and **4** were prepared by reaction of [Cu(MeCN)_4_][PF_6_] with two equivalents of ligand in a mixture of CH_2_Cl_2_ and CH_3_CN. The dark red products were precipitated from MeCN by addition of Et_2_O, and were isolated in yields of between 33.1% and 72.9%. The high-resolution electrospray mass spectra of the compounds exhibit peaks arising from the [Cu(**L**)_2_]^+^ ions with isotope distributions that agree with those predicted (Figures S17–S20 in Supporting Information). The IR spectra of the compounds exhibit a strong absorption at 838 cm^−1^ for [Cu(**1**)_2_][PF_6_], 842 cm^−1^ for [Cu(**2**)_2_][PF_6_], 831 cm^−1^ for [Cu(**3**)_2_][PF_6_], and 833 cm^−1^ for [Cu(**4**)_2_][PF_6_], characteristic of the [PF_6_]^−^ ion, and for [Cu(**2**)_2_][PF_6_] and [Cu(**4**)_2_][PF_6_], absorptions at 2189 and 2200 cm^−1^ are consistent with the presence of the alkynyl unit (Figures S21–S24). The ^1^H and ^13^C{^1^H} NMR spectra were assigned using NOESY, COSY, HMQC and HMBC methods (Figures S25–S36). Coordination to copper(I) results in a broadening of the methyl signal (Figure 2), as well as shifting of the signal arising from proton H^A5^.

**Figure 3:**
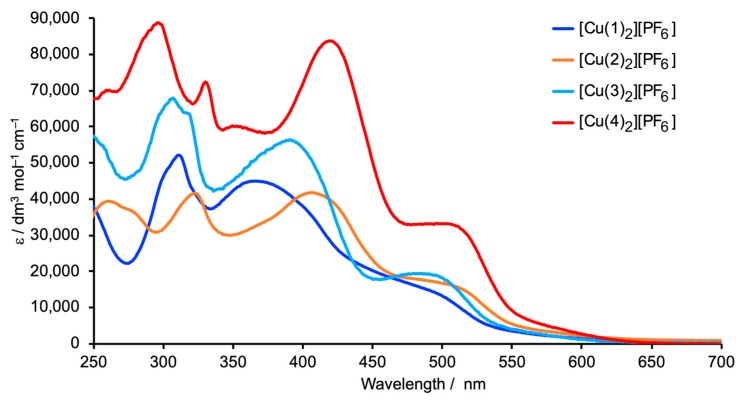
Solution absorption spectra of [CuL_2_][PF_6_] with L = **1**–**4** (CHCl_3_, 3.4 × 10^−5^ mol dm^−3^ for L = **1**, 1.0 × 10^−5^ mol dm^−3^ mol dm^−3^ for L = **2**–**4**).

The solution absorption spectra of the complexes were recorded in CHCl_3_ (Figure 3) and exhibit intense, high-energy absorptions arising from ligand-centred, spin-allowed transitions in addition to absorptions in the visible region. The lowest energy absorption around 500 nm is assigned to metal-to-ligand charge-transfer (MLCT). However, the band exhibits a higher extinction coefficient than is typical of bis(diimine)copper(I) complexes [19,46] due to overlap with the ligand-centred ILCT observed in the free ligands (Figure 2). Inspection of Figure 3 reveals that the intensity of the absorption at ca. 500 nm is greatest for [Cu(**3**)_2_][PF_6_] and [Cu(**4**)_2_][PF_6_], indicating that the Ph_2_N group plays a role. We also note that on going from [Cu(**1**)_2_][PF_6_] to [Cu(**2**)_2_][PF_6_], and from [Cu(**3**)_2_][PF_6_] to [Cu(**4**)_2_][PF_6_], the absorption spectra extends further towards the red, consistent with extension of π-conjugation as the alkynyl unit is introduced.

**Figure 4:**
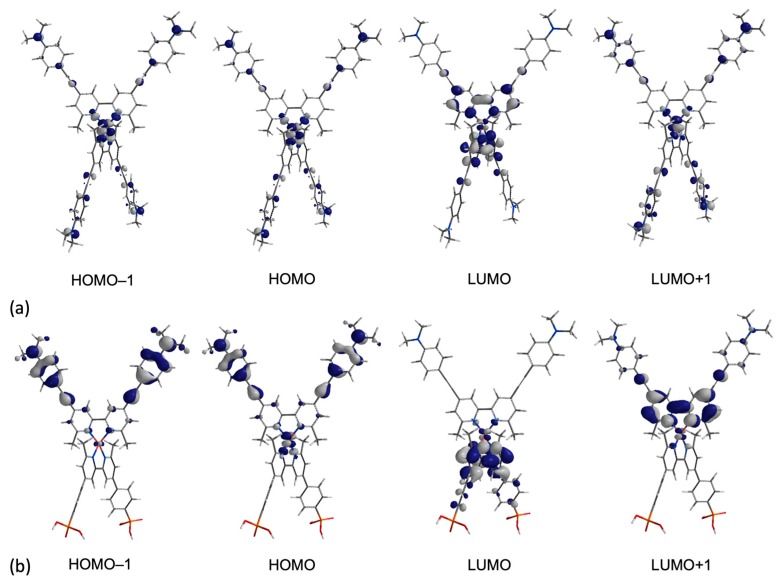
Orbital compositions of the highest occupied and lowest unoccupied molecular orbitals in (**a**) [Cu(**2**)_2_]^+^ and (**b**) [Cu(**5**)(**2**)]^+^ calculated using DFT (B3LYP level, 6-31G* basis set, in vacuum). Geometry optimizations were at the same DFT level of calculation.

**Figure 5:**
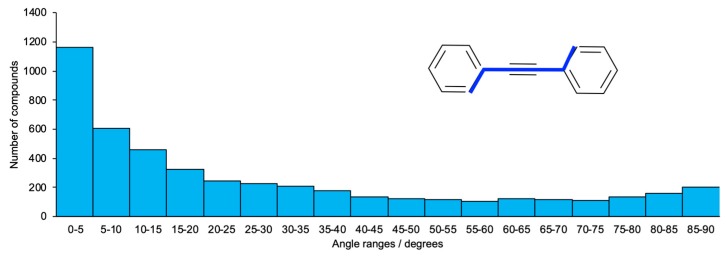
Distribution of torsion angles (defined in the inserted structure and considered for a range 0–90^o^) for alkynyl-connected phenyl rings in the Cambridge Structural Database (CSD v. 5.4.1), with analysis using Conquest, version 2.0.4 [49].

DFT calculations on [Cu(**2**)_2_]^+^ as a representative complex confirmed that the orbital composition in the HOMO-manifold contains both metal and Me_2_N character, while the LUMO and LUMO+1 are predominantly bpy character (Figure 4a). A restricted hybrid HF-DFT SCF calculation was also performed on [Cu(**2**)_2_]^+^ using a polarizable continuum solvation model (CHCl_3_) and revealed similar frontier molecular orbital ( MO) characteristics (Figure S37). Note that the DFT-optimized geometries reveal that in ligand **1**, the phenyl ring is twisted with respect to the pyridine ring to which it is bonded, as expected for minimizing inter-ring H...H contacts. In contrast, in **2**, the aromatic rings on either side of the alkynyl unit are essentially coplanar. This is consistent with previously reported theoretical results [47] and with the distribution of dihedral angles between alkynyl-connected phenyl rings for compounds found in the Cambridge Structural Database (CSD v. 5.4.1 [48]) (Figure 5).

### 2.3. Solid-State Absorption Spectra of the Surface-Bound Dyes and DSC Performances

The homoleptic compounds [CuL_2_][PF_6_] where L = **1**–**4**, were used as a source of copper(I) and ancillary ligand **1**–**4** to assemble surface-bound heteroleptic dyes [Cu(**5**)(L)]^+^ using the surfaces-as- ligands, surfaces-as-complex (SALSAC) ligand exchange methodology [50,51]. The phosphonic acid anchoring ligand **5** (Scheme 2) was selected because of its superior binding to TiO_2_ with respect to carboxylic acids [51], and because copper(I) dyes with phosphonic acids or phosphonates [52] with a phenyl spacer between the PO(OH)_2_ and bpy units are superior to carboxylic and cyanoacrylic acids in terms of DSC performance and/or ease of synthesis [53,54,55].

**Figure 6:**
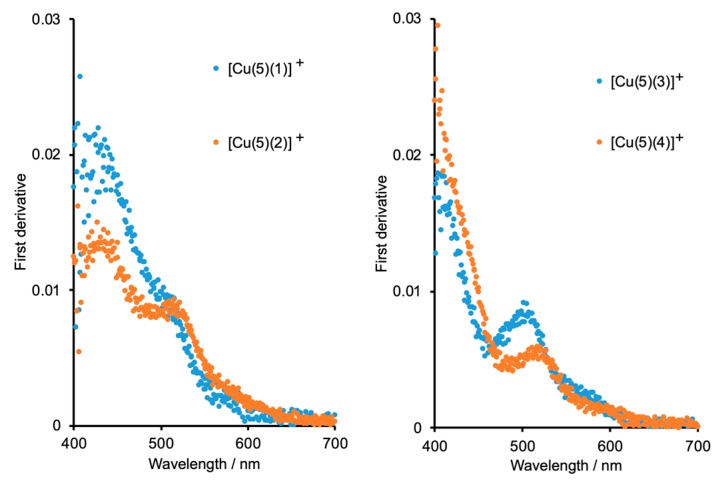
First-derivative solid-state absorption spectra of transparent FTO/TiO_2_ electrodes functionalized with the [Cu(**5**)(L)]^+^ dyes (L = **1**, **2**, **3** and **4**).

The solid-state absorption spectra of the heteroleptic dye-functionalized FTO/TiO_2_ electrodes were recorded. In order to better differentiate the absorption maxima in the visible region from the higher-energy tail arising from TiO_2_, the first-derivative spectra [56] were determined (Figure 6). The maxima at around 510 nm in [Cu(**5**)(**1**)]^+^ and [Cu(**5**)(**3**)]^+^, and 520 nm in [Cu(**5**)(**2**)]^+^ and [Cu(**5**)(**4**)]^+^ confirm that a red-shift in the absorptions accompanies the introduction of the alkyne spacer in the heteroleptic compounds. Absorption maxima for the surface-bound dyes compare with solution values of 500 nm and 493 nm in [Cu(**1**)_2_][PF_6_] and [Cu(**3**)_2_][PF_6_], respectively, and 515 nm and 507 nm in [Cu(**2**)_2_][PF_6_] and [Cu(**4**)_2_][PF_6_], respectively (Figure 3).

DFT calculations on [Cu(**5**)(**2**)]^+^ show that the orbital compositions of the HOMO and HOMO–1 contain both metal and ancillary-ligand character, while the LUMO is localized on the anchoring ligand **5** (Figure 4b); the LUMO + 1 exhibits mainly ancillary ligand bpy character. Similar orbital characters are revealed for [Cu(**5**)(**1**)]^+^ (Figure S38 in the Supporting Information), indicating that the frontier orbital characteristics of the heteroleptic dyes are not significantly perturbed by the presence of the alkynyl spacers. Thus, the localization of LUMO character on the anchoring domain is consistent with what is desired for efficient electron injection from the anchoring ligand to the semiconductor in a DSC.

**Figure 7:**
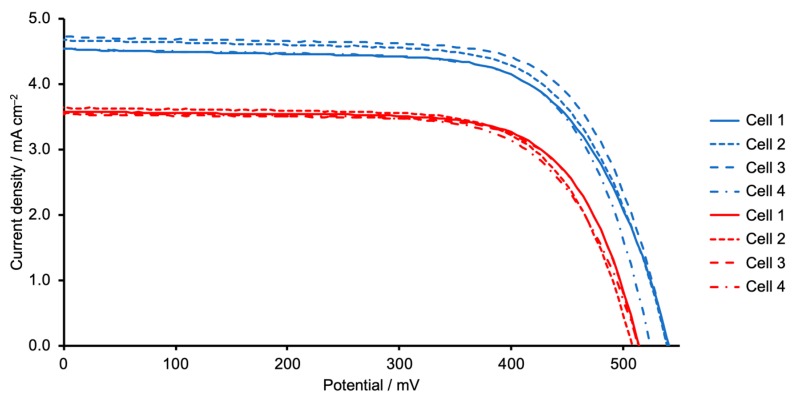
*J–V* (*J* = current density, *V* = voltage) curves for sets of four dye-sensitized solar cells (DSCs) sensitized with [Cu(**5**)(**1**)]^+^ (blue curves) and [Cu(**5**)(**2**)]^+^ (red curves) measured on the day of sealing the cells.

**Figure 8:**
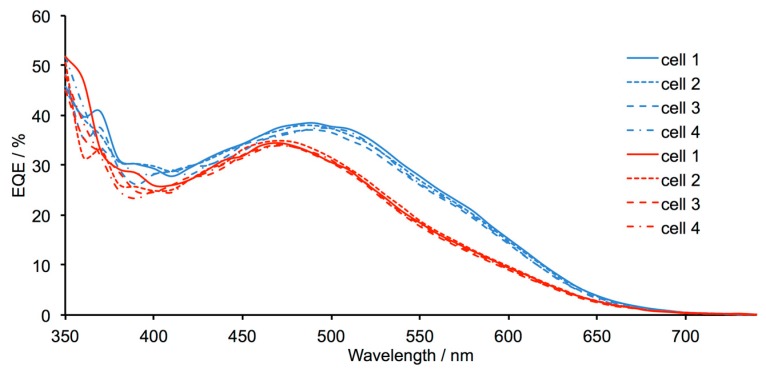
External quantum efficiency (EQE) spectra for sets of four DSCs sensitized with [Cu(**5**)(**1**)]^+^ (blue curves) and [Cu(**5**)(**2**)]^+^ (red curves) measured on the day of sealing the cells.

Table 1 summarizes the performances of sets of four DSCs sensitized with the dyes [Cu(**5**)(**1**)]^+^ and [Cu(**5**)(**2**)]^+^ compared with the performance of a DSC containing the reference ruthenium(II) dye N719. In the final column of Table 1, we present relative efficiency values with N719 set at an arbitrary 100%. We [57] and others [58,59] find it useful to include relative values so as to permit valid comparisons of data recorded on different solar simulators or in different laboratories [57]. The first point to note in Table 1 is the reproducibility of the DSC performance parameters for a given sensitizer. This is also observed in the *J–V* (*J* = current density, *V* = voltage) plots in Figure 7 and Figure S39. The most noticeable feature is that the DSCs containing [Cu(**5**)(**2**)]^+^ have both lower *J*_SC_ and *V*_OC_ values than those with [Cu(**5**)(**1**)]^+^, indicating that the introduction of the alkynyl spacer in **2** is not beneficial. The PCEs for DSCs sensitized with [Cu(**5**)(**1**)]^+^ are in the range 1.66–1.79%, which corresponds to 31.0–33.4% of the PCE of the reference DSC containing the ruthenium(II) dye N719. Figure 8 and Figure S40 show the external quantum efficiency (EQE) spectra for the cells in Table 1, with values of *λ*_max_ ≈ 490 nm and EQE_max_ ≈ 38% for [Cu(**5**)(**1**)]^+^, and *λ*_max_ ≈ 470 nm and EQE_max_ ≈ 34% for [Cu(**5**)(**2**)]^+^. These data are consistent with the differences in the *J–V* curves for the two dyes.

**Scheme 6:**
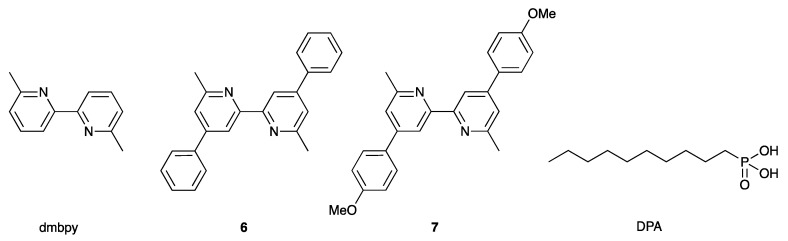
Structures of ancillary ligands dmbpy, **6**, and **7**, and the co-adsorbent *n*-decylphosphonic acid (DPA).

**Figure 9:**
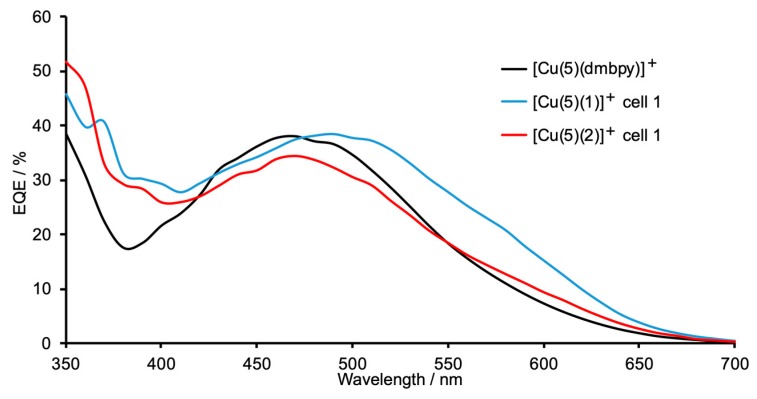
A comparison of the EQE spectra for DSCs sensitized with [Cu(**5**)(**1**)]^+^ (blue curve), [Cu(**5**)(**2**)]^+^ (red curve) and [Cu(**5**)(dmbpy)]^+^ (black curve, data from ref [57]), measured on the day of sealing the cells.

A critical question is why the introduction of the alkynyl spacer into the dye leads to a blue-shifted EQE maximum (Figure 8) rather than the red-shift observed in the absorption spectra (Figure 3 and Figure 6). This reveals that the injection of electrons into the semiconductor is not benefitting from an extension of light absorption towards longer wavelengths. It is informative to compare the performance data and EQE spectra for DSCs with [Cu(**5**)(**1**)]^+^ and [Cu(**5**)(**2**)]^+^ with those sensitized with [Cu(**5**)(dmbpy)]^+^ where dmbpy is 6,6′-dimethyl-2,2′-bipyridine (Scheme 6). Using a common electrolyte, redox shuttle and sun simulator as employed in the present study (see Materials and Methods section), duplicate DSCs with [Cu(**5**)(dmbpy)]^+^ exhibited values of *J*_SC_ = 3.46 and 3.79 mA cm^−2^, *V*_OC_ = 522 and 527 mV, and *η* = 1.46% and 1.35% versus 5.91% for N719 (i.e., relative *η* = 24.7% and 22.8%). The EQE_max_ values were 42% and 38% with a value of *λ*_max_ = 470 nm [57]. These data are reminiscent of those for DSCs containing [Cu(**5**)(**2**)]^+^. We therefore see an enhancement of DSC performance on going from the dye [Cu(**5**)(dmbpy)]^+^ to [Cu(**5**)(**1**)]^+^, but not from [Cu(**5**)(dmbpy)]^+^ to [Cu(**5**)(**2**)]^+^. The comparison of EQE spectra shown in Figure 9 illustrates a gain in EQE arising from photon harvesting at longer wavelengths on going from [Cu(**5**)(dmbpy)]^+^ to [Cu(**5**)(**1**)]^+^, but this enhancement is lost once the alkynyl spacer is introduced with the EQE spectrum of the DSC with [Cu(**5**)(**2**)]^+^ looking very similar to that of the cell with [Cu(**5**)(dmbpy)]^+^. An additional comparison can be made between the EQE spectra of DSCs sensitized with the dyes [Cu(**5**)(**6**)]^+^ and [Cu(**5**)(**7**)]^+^ (see Scheme 6 for the structures of **6** and **7**), [Cu(**5**)(**1**)]^+^ and [Cu(**5**)(**2**)]^+^. The value of *λ* corresponding to EQE_max_ is 470 nm for [Cu(**5**)(**6**)]^+^ [60], the same as for [Cu(**5**)(dmbpy)]^+^ and [Cu(**5**)(**2**)]^+^. Introducing electron-donating 4-methoxy groups on going from ancillary ligand **6** to **7** shifts *λ*_max_ in the EQE spectrum of DSCs with [Cu(**5**)(**7**)]^+^ to 480 nm [60], consistent with the red-shift seen on going from [Cu(**5**)(**6**)]^+^ to [Cu(**5**)(**1**)]^+^. The photoconversion efficiencies of DSCs with [Cu(**5**)(**1**)]^+^ (peripheral NMe_2_ groups, Table 1) are similar to those sensitized with [Cu(**5**)(**7**)]^+^ (peripheral OMe groups [60]); the electrolyte and redox shuttle are constant throughout. The comparison of this series of dyes reveals the beneficial effects of introducing the electron-donating, peripheral substituents, but the ‘blocking’ effect that the alkynyl group imparts on electron transfer and ultimate injection.

**Figure 10:**
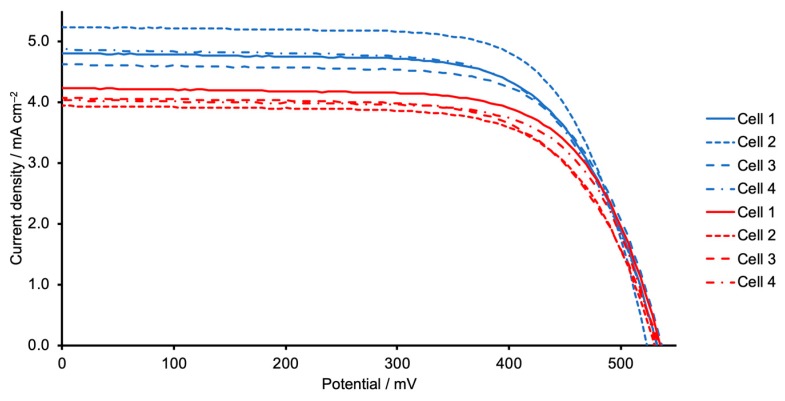
*J–V* curves for sets of four DSCs sensitized with [Cu(**5**)(**3**)]^+^ (blue curves) and [Cu(**5**)(**4**)]^+^ (red curves) measured on the day of sealing the cells.

**Figure 11:**
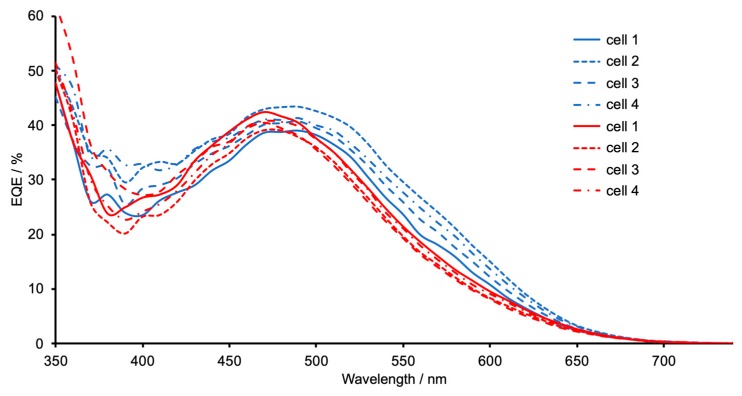
EQE spectra for sets of four DSCs sensitized with [Cu(**5**)(**3**)]^+^ (blue curves) and [Cu(**5**)(**4**)]^+^ (red curves) measured on the day of sealing the cells.

A similar trend in DSC performances is observed for dyes [Cu(**5**)(**3**)]^+^ versus [Cu(**5**)(**4**)]^+^ as for [Cu(**5**)(**1**)]^+^ versus [Cu(**5**)(**2**)]^+^. Introduction of the alkynyl unit on going from **3** to **4** leads to lower *J*_SC_ values although values of *V*_OC_ are little affected (Figure 10 and Table 2). The change from Me_2_N (ligands **1** and **2**) to Ph_2_N (ligands **3** and **4**) substituents results in a small improvement in DSC performance, and this is more noticeable on going from [Cu(**5**)(**2**)]^+^ to [Cu(**5**)(**4**)]^+^ than from [Cu(**5**)(**1**)]^+^ to [Cu(**5**)(**3**)]^+^ (Table 1 and Table 2). This has its origins in a small increase in *J*_SC_, which, for four cells, lies in the range of 3.34–3.64 mA cm^−2^ for [Cu(**5**)(**2**)]^+^, and 3.96–4.24 mA cm^−2^ for [Cu(**5**)(**4**)]^+^. This trend is confirmed in the EQE spectra (Figure 10), which extend further to longer wavelengths for DSCs containing [Cu(**5**)(**4**)]^+^. As was observed for cells with dyes [Cu(**5**)(**1**)]^+^ and [Cu(**5**)(**2**)]^+^, values of *λ*_max_ in the EQE spectra for [Cu(**5**)(**3**)]^+^ and [Cu(**5**)(**4**)]^+^ are around 470 nm and 490 nm, respectively. A comparison of Figure 8 and Figure 11 reveals an increase in EQE_max_ for [Cu(**5**)(**4**)]^+^ (up to 42%) compared to [Cu(**5**)(**2**)]^+^ (≈34%) as well as a significant extension of the spectrum for [Cu(**5**)(**4**)]^+^ towards the red. These data are consistent with the improvement in *J*_SC_ as the peripheral electron-donating NMe_2_ substituents are replaced by NPh_2_ groups.

### 2.4. Effects of Adding a Co-Adsorbent

It is well established that introducing co-adsorbents with dyes on the semiconductor surface increases values of *V*_OC_ for ruthenium(II) dyes [61] and zinc(II) porphyrin dyes [62,63]. Similarly, the role of the co-adsorbent chenodeoxycholic acid was critical to achieving the high PCE of the [Cu(L_anchor_)(L_ancillary_)]^+^ dye in which L_anchor_ was 6,6′-dimesityl-2,2′-bipyridine-4,4′-dicarboxylic acid and L_ancillary_ was a 2,2′-bipyridine containing electron-donating triphenylamino groups [21]. We were therefore interested to see whether use of a co-adsorbent could enhance the performance of the dyes in the present investigation, and we chose to investigate the dye [Cu(**5**)(**1**)]^+^. Chenodeoxycholic acid (cheno) is commonly employed as a co-adsorbent and contains a carboxylic acid anchoring unit. In contrast, anchoring ligand **5** bears a phosphonic acid group. Since we have shown that phosphonic anchoring ligands displace carboxylic anchors in TiO_2_ [51], we opted to use *n*-decylphosphonic acid (DPA, Scheme 6) instead of cheno as the co-adsorbent.

**Figure 12:**
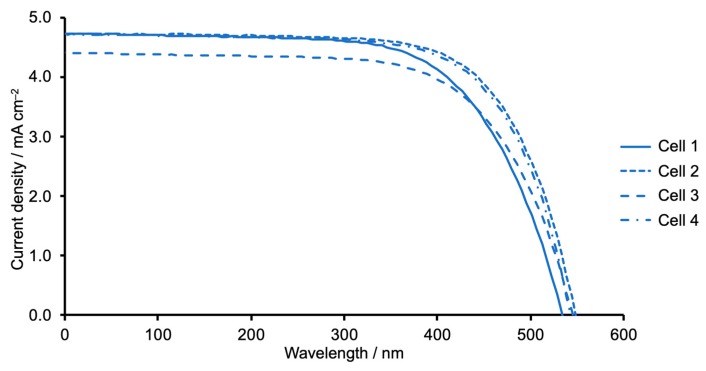
*J–V* curves for a set of four DSCs sensitized with [Cu(**5**)(**1**)]^+^ with DPA as a co-adsorbent, measured on the day of sealing the cells.

**Figure 13:**
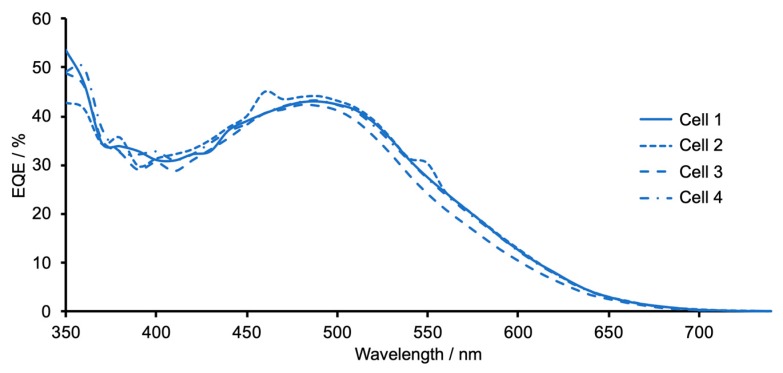
EQE spectra for a set of four DSCs sensitized with [Cu(**5**)(**1**)]^+^ with DPA as a co-adsorbent, measured on the day of sealing the cells.

Table 3 gives the DSC performance parameters for four DSCs sensitized with [Cu(**5**)(**1**)]^+^ in the presence of the co-adsorbent DPA, and the *J–V* curves for the cells are shown in Figure 12. The ranges of *J*_SC_ (4.42 to 4.75 mA cm^−2^) and *J*_SC_ values (534 to 548 mV) are similar to those for cells with no co-adsorbent (Table 1) leading to similar overall photoconversion efficiencies. The EQE spectra (Figure 13) confirm reproducible behaviour for the four DSCs with values of EQE_max_ around 43% at a value of *λ*_max_ = 490 nm. Again, these results reflect those of the devices in the absence of DPA. Evidence that the co-adsorbent is present on the semiconductor surface comes from the electrochemical impedance spectroscopic data presented below.

### 2.5. Electrochemical Impedance Spectroscopy (EIS)

**Figure 14:**
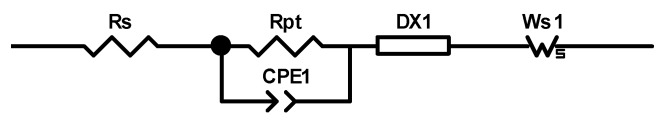
Equivalent circuit used to fit the electrochemical impedance spectroscopy (EIS) data, where *R*_s_ is the series resistance, *R*_Pt_ and CPE1 are the resistance and constant phase element of the platinum counter electrode, DX1 is an extended distributed element that represents the TiO_2_/electrolyte interface and *W*_S_ is the Warburg element associated with diffusion of the electrolyte.

As discussed above, the introduction of the alkynyl spacer into the dyes results in a red-shifted absorption (Figure 3 and Figure 6), but a blue-shifted EQE maximum (Figure 8) as well as a decrease of EQE_max_. The lowering of the charge transfer energy results in more difficult electron injection into the semiconductor, and the values of *J*_SC_ decrease. By using electrochemical impedance spectroscopy (EIS), it is possible to investigate the multiple interfacial electronic processes in a DSC [64,65] and we therefore decided to apply EIS in an attempt to gain insight into the origin of the poorer DSC performances for dyes containing the alkynyl spacers. EIS is a technique that can describe electronic processes in terms of electron and hole diffusion in the counter electrode/electrolyte/dye-semiconductor interfaces of DSCs. Parameters including the recombination resistance (R_rec_), chemical capacitance (C_µ_), electron/hole transport resistance (R_tr_), electron lifetime (τ) and electron diffusion length (L_d_) can be extracted from the fits of the experimental data typically presented as Nyquist and Bode plots. All measurements were performed at *V*_OC_ conditions and a light intensity of 22 mW cm^−2^. The equivalent circuit model used for fitting the measurements is shown in Figure 14.

**Figure 15:**
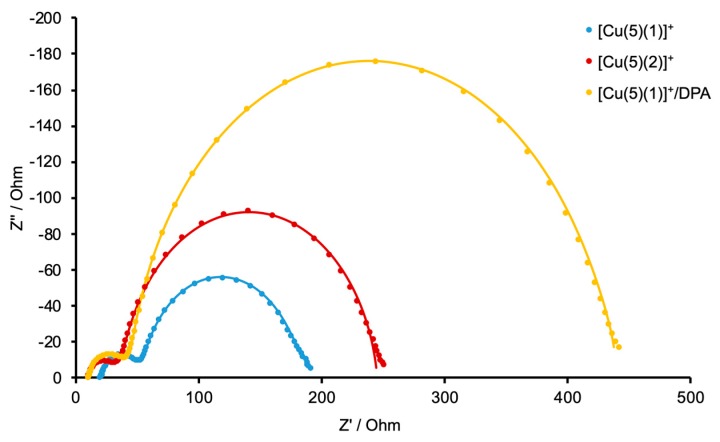
Nyquist plots of the DSCs containing dyes [Cu(**5**)(**1**)]^+^ and [Cu(**5**)(**2**)]^+^, and [Cu(**5**)(**1**)]^+^ with the co-adsorbent DPA. Solid lines represent fitted curves and the solid-circles represent experimental data.

We first focused our attention on a comparison of DSCs sensitized with [Cu(**5**)(**1**)]^+^ and [Cu(**5**)(**2**)]^+^, and parameters extracted from fitting of the experimental Nyquist plots (Figure 15) are given in Table S1 (all data) and Table 4 (the most representative DSC of a set of four). Going from the DSCs with [Cu(**5**)(**1**)]^+^ to those [Cu(**5**)(**2**)]^+^, the values of *R*_rec_ increase. At the same time, values of *C*_µ_ are higher for [Cu(**5**)(**1**)]^+^ than [Cu(**5**)(**2**)]^+^. This shows that more electron density is located in the conductive band of the semiconductor in the case of the dye [Cu(**5**)(**1**)]^+^, which contributes to greater *J*_SC_ values. The short-circuit density depends not only on the charge injection, but also on the collection of photoinjected electrons at the anode [66], and the electron diffusion length has to be greater than thickness of TiO_2_ for the efficient collection of electrons. The *L*_d_ value depends on *R*_rec_ and *R*_tr_ and can be calculated from the Equation (1) where *d* (≈ 12 μm) is the thickness of the TiO_2_ layer [67].

(1) $$Ld=d\sqrt{Rrec/Rtr}$$

The diffusion length does not vary significantly between the dyes [Cu(**5**)(**1**)]^+^ and [Cu(**5**)(**2**)]^+^ and is about two times greater than *d*, despite the difference in EQE_max_ values and red-shifted enhancement in the range of the spectrum for [Cu(**5**)(**1**)]^+^. The addition of the co-adsorbent DPA in the DSCs with dyes [Cu(**5**)(**1**)]^+^ results in a significant difference in EQE_max_ as well as in *L*_d_. The diffusion length and EQE_max_ values for [Cu(**5**)(**1**)]^+^ with co-adsorbent increase with respect to [Cu(**5**)(**1**)]^+^ with no co-adsorbent. However, this in accompanied by a decrease in *C*_µ_ and a large increase in *R*_rec_ and this results in similar overall performances for DSCs with and without co-adsorbent.

**Figure 16:**
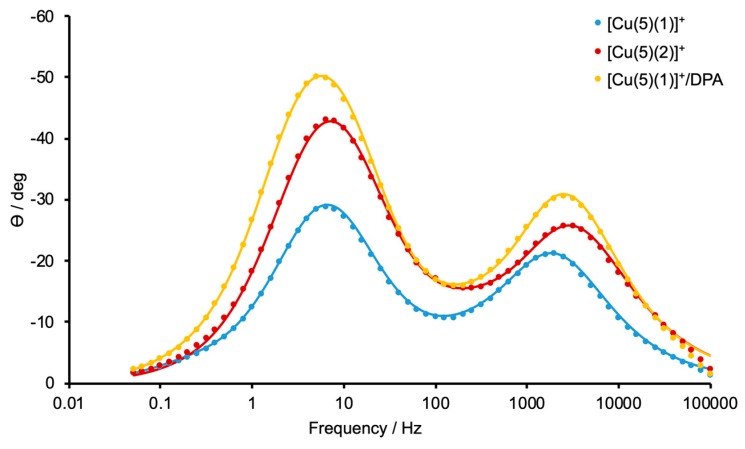
Bode plots of the DSCs containing dyes [Cu(**5**)(**1**)]^+^ and [Cu(**5**)(**2**)]^+^, and [Cu(**5**)(**1**)]^+^ with the co-adsorbent DPA. Solid lines represent fitted curves and the solid-circles represent experimental data.

For a well-performing DSC, the electron lifetime (*τ*) needs to be longer than electron transport time (*τ*_t_). This results in the efficient transport of the photoinjected electrons through the semiconductor, and this is the case for all the DSCs (Table 4 and Table S1). The extremely high *τ* for [Cu(**5**)(**1**)]^+^ with a co-adsorbent (range of values = 79–99 ms for four cells) is observed due to the high *R*_rec_ values for this dye (range = 293–384 Ω). The trend in lifetime values can be confirmed from the Bode plots (Figure 16) since *τ* is related to the maximum frequency. A high *τ* in combination with increased *L*_d_ values result in a small charge loss in the semiconductor. The parameters for the counter electrode stay constant for all DSCs.

The most significant differences in EIS parameters are observed for values of the recombination resistance and chemical capacitance, and we conclude that [Cu(5)(1)]^+^ has the most favourable electron injection into the conduction band of the semiconductor.

## 3. Materials and Methods

^1^H and ^13^C and NMR spectra were recorded on a Bruker Avance iii-500 spectrometer (Bruker BioSpin AG, Fällanden, Switzerland) at 298 K. The ^1^H and ^13^C NMR chemical shifts were referenced with respect to residual solvent peaks (*δ* TMS = 0). A Shimadzu LCMS-2020 and a Bruker maXis 4G QTOF instrument (Bruker BioSpin AG, Fällanden, Switzerland) were used to record electrospray ionization (ESI) and HR-ESI mass spectra, respectively. FT-infrared (IR) and absorption spectra were measured using PerkinElmer UATR Two (Perkin Elmer, 8603 Schwerzenbach, Switzerland), Cary-5000 (Agilent Technologies Inc., Santa Clara, CA, United States) and UV-2600 (Shimadzu Schweiz GmbH, 4153 Reinach, Switzerland) spectrophotometers.

All reactions were carried out with chemicals used as received from Sigma Aldrich (Sigma Aldrich Chemie GmbH, 89555 Steinheim, Germany) or Fluorochem (Chemie Brunschwig AG, 4052 Basel, Switzerland) without further purification. Biotage Sfär silica HC D was purchased from Biotage (Biotage EU, 75103 Uppsala, Sweden).

Compound **5** [53], *N,N*-dimethyl-4-((trimethylsilyl)ethynyl)aniline [68], 4-ethynyl-*N,N*-dimethylaniline [69], 4,4′-dibromo-6,6′-dimethyl-2,2′-bipyridine [70] and [Cu(MeCN)_4_][PF_6_] [71] were prepared as previously reported in literature.

### 3.1. (1E,5E)-1,6-Bis(4-(dimethylamino)phenyl)hexa-1,5-diene-3,4-dione

4-(Dimethylamino)benzaldehyde (2.24 g, 15 mmol, 2.0 eq) and piperidine (148 µL, 1.5 mmol, 0.2 eq) were dissolved in MeOH (15 mL). Butan-2,3-dione (656 mL, 7.5 mmol, 1.0 eq) was dissolved in in MeOH (12 mL) and added to the reaction over 10 min. The reaction mixture was stirred for 1.5 h and then heated under reflux overnight. The precipitate that formed was separated by filtration, washed with Et_2_O and dried under vacuum. The product was isolated as a red solid (411 mg, 1.18 mmol, 15.7%). Decomposes > 248 °C. ^1^H NMR (500 MHz, CDCl_3_): *δ/*ppm 7.79 (d, *J* = 15.9 Hz, 2H, H^b^), 7.55 (d, *J* = 8.8 Hz, 4H, H^B2^), 7.23 (d, *J* = 15.9 Hz, 2H, H^a^), 6.71 (d, *J* = 8.5 Hz, 4H, H^B3^), 3.06 (s, 12H, H^NMe^). ^13^C{^1^H} NMR (126 MHz, CDCl_3_): *δ*/ppm 190.3 (C^CO^), 152.1 (C^B4^), 148.2 (C^b^), 131.1 (C^B2^), 123.0 (C^B1^), 115.5 (C^a^), 112.2 (C^B3^), 40.4 (C^NMe^). UV-VIS (CHCl_3_, 3.3 × 10^−5^ mol dm^−3^) λ/nm 262 (ε/dm^3^ mol^−1^ cm^−1^ 12,500), 327 (ε/dm^3^ mol^−1^ cm^−1^ 14,600), 348 (ε/dm^3^ mol^−1^ cm^−1^ 14,800), 364 (ε/dm^3^ mol^−1^ cm^−1^ 14,059), 454 (ε/dm^3^ mol^−1^ cm^−1^ 43,700). HRMS *m*/*z* 349.1905 [M + H]^+^ (calc. 349.1911).

### 3.2. 4,4′-Bis(4-dimethylaminophenyl)-6,6′-dimethyl-2,2′-bipyridine (***1***)

1-(2-Oxopropyl)pyridin-1-ium chloride (1437 mg, 8.38 mmol, 2.5 eq) was dissolved in EtOH (150 mL) under vigorous stirring. The (1*E*,5*E*)-1,6-Bis(4-(dimethylamino)phenyl)hexa-1,5-diene-3,4-dione (1167 mg, 3.35 mmol, 1.0 eq.) and ammonium acetate (3873 mg, 50.3 mmol, 15 eq.) were then added followed by additional EtOH (30 mL). The reaction mixture was heated under reflux overnight, after which it was cooled to room temperature while stirring. The precipitate that had formed was separated by filtration, washed with cold MeOH and then cold Et_2_O. The product was recrystallized from *N,N*-dimethylformamide (DMF) and after filtration the product was washed with cold EtOH and dried. The compound was isolated as a fine yellow powder (131 mg, 0.31 mmol, 9.25%). Decomposes > 248 °C. ^1^H NMR (500 MHz, CDCl_3_): *δ*/ppm 8.51 (s, 2H, H^A3^), 7.76 (d, *J* = 8.7 Hz, 4H, H^B2^), 7.47 (s, 2H, H^A5^), 6.81 (d, *J* = 8.7 Hz, 4H, H^B3^), 3.05 (s, 12H, H^NMe^), 2.76 (s, 6H, H^A6-Me^). ^13^C{^1^H} NMR (126 MHz, CDCl_3_): *δ*/ppm 157.5 (C^A6^), 151.6 (C^B4^), 128.7 (C^B2^), 124.5 (C^B1^), 120.6 (C^A5^), 117.2 (C^A3^), 112.7 (C^B3^), 40.7 (C^NMe^), 23.8 (C^A6-Me^); C^A2^, C^A4^ not resolved in HMBC. UV-VIS (CHCl_3_, 2.5 × 10^−5^ mol dm^−3^) λ/nm 291 sh (ε/dm^3^ mol^−1^ cm^−1^ 28,800), 336 (42,700), 429 (2,300). HRMS *m*/*z* 423.2544 [M + H]^+^ (calc. 423.2543). IR spectrum: see Figure S1 in the Supporting Information.

### 3.3. 4,4′-Bis(4-diphenylaminophenyl)-6,6′-dimethyl-2,2′-bipyridine (***3***)

4,4′-Bis(4-bromophenyl)-6,6′-dimethyl-2,2′-bipyridine (100 mg, 203 µmol, 1.0 eq), diphenylamine (103 mg, 609 µmol, 3.0 eq), Pd(*^t^*Bu_3_P)_2_ (4.15 mg, 8.12 µmol, 4 mol%), and NaO*^t^*Bu (64.4 mg, 670 µmol, 3.3 eq) were loaded into a heat-dried microwave vial, previously degassed and purged with N_2_ three times. After a further 15 min of N_2_ purging, toluene (6.9 mL) was added to the mixture, and the mixture was stirred at 100 °C overnight. After cooling to room temperature, the reaction mixture was separated from the solid residues with a Pasteur pipette, then dried under reduced pressure. The residue was then boiled in EtOH (25 mL ca.) and the organic layer was then separated from the suspension by decantation and removal with Pasteur pipette. The remaining solid was then boiled in CHCl_3_ (25 mL ca.), filtered through a Celite plug (to remove Pd dust), and finally removed under reduced pressure. The product was isolated as a sticky yellow-brown solid (97.3 mg, 145 µmol, 71.4%). ^1^H NMR (500 MHz, CDCl_3_): *δ*/ppm 8.52 (br, 2H, H^A3^), 7.68 (br d, 4H, H^B2^), 7.40 (br, 2H, H^A5^), 7.30 (t, *J* = 7.7 Hz, 8H, H^C3^), 7.15 (overlapping m, 12H, H^C2+B3^), 7.08 (t, *J* = 7.2 Hz, 4H, H^C4^), 2.74 (s, 6H, H^Me^). ^13^C{^1^H} NMR (126 MHz, CDCl_3_): *δ/*ppm 147.3 (C^C1^), 132.0 (C^B1^), 129.5 (C^C3^), 128.1 (C^B2^), 124.9 (C^C2^), 123.4 (C^C4^), 123.0 (C^B3^), 120.4 (C^A5^), 116.5 (C^A3^), 24.6 (C^Me^), signals for C^A2^, C^A4^, C^A6^, C^B4^ not resolved. UV-VIS (CHCl_3_, 2.5 × 10^−5^ mol dm^−3^) λ/nm 308 sh (ε/dm^3^ mol^−1^ cm^−1^ 30,000), 362 (51,000), 457 (4700). HRMS *m*/*z* 671.3166 [M + H]^+^ (calc. 671.3169). IR spectrum: see Figure S3 in the Supporting Information.

### 3.4. N,N-Diphenyl-4-((trimethylsilyl)ethynyl)aniline

*N,N*-Diphenyl-4-((trimethylsilyl)ethynyl)aniline was prepared in the same manner as *N,N*-dimethyl-4-((trimethylsilyl)ethynyl)aniline [68] rather than by the previously described method [72]. 4-Bromophenylamine (1.002 g, 3.09 mmol, 5.0 eq), Pd(dppf)Cl_2_ (136 mg, 185 µmol, 6 mol%), CuI (58.8 mg, 309 µmol, 0.5 eq) and PPh_3_ (81.0 mg, 309 µmol, 0.5 eq) were loaded in a 10 mL microwave vial, degassed and purged with N_2_ three times. After 15 min of N_2_ purging, 2 mL of Et_3_N were added to the mixture and set under stirring. Finally, trimethylsilylethyne (910 mg, 9.27 µmol, 15 eq) was added to the reaction mixture and was set at 80 °C overnight. After cooling down to room temperature, the mixture was poured in water (10 mL) and extracted with AcOEt (3 × 10 mL), the organic layers were washed further with brine (30 mL) and back-extracted. The organic layers were collected together, dried over MgSO_4_, and dried under reduced pressure. The crude mixture was purified by column chromatography (Biotage Sfär silica HC D, petroleum ether/CH_2_Cl_2_, CH_2_Cl_2_ increasing gradient up to 10%). The product was isolated as a brown solid (1.027 g, 3.007 mmol, 97.30%). ^1^H NMR (500 MHz, CDCl_3_): *δ*/ppm 7.33 (d, *J* = 8.7 Hz, 2H), 7.27 (m, 4H), 7.08 (m, 4H), 7.06 (m, 2H), 6.95 (d, *J* = 8.7 Hz, 2H), 0.26 (s, 9H) as reported in the literature [73].

### 3.5. 4-Ethynyl-N,N-diphenylaniline

Deprotection of *N,N*-diphenyl-4-((trimethylsilyl)ethynyl)aniline to give 4-ethynyl-*N,N*-diphenylaniline was carried out as previously described [72]. ^1^H NMR (500 MHz, CDCl_3_): *δ/*ppm 7.32 (m, 2H), 7.26 (m, 4H), 7.09 (d, *J* = 7.7 Hz, 2H), 7.06 (t, *J* = 7.6 Hz, 2H), 6.96 (m, 2H), 3.01 (s, 1H).

### 3.6. 4,4′-Bis(4-dimethylaminophenylethynyl)-6,6′-dimethyl-2,2′-bipyridine (***2***)

4,4′-Dibromo-6,6′-dimethyl-2,2′-bipyridine (150 mg, 439 µmol, 1.0 eq), 4-ethynyl-*N,N*-dimethylaniline (159 mg, 1.1 mmol, 2.5 eq), Pd(dppf)Cl_2_ (19.3 mg, 26.3 µmol, 6 mol%), CuI (8.36 mg, 43.9 µmol, 10 mol%), and PPh_3_ (11.5 mg, 43.9 µmol, 0.1 eq) were loaded into a 5 mL microwave vial, previously degassed and purged with N_2_ three times. After 15 min of N_2_ purging, Et_3_N (875 µL) was added to the reaction mixture, which was then stirred at 80 °C overnight. After cooling to room temperature, the reaction mixture was poured into water (10 mL) and extracted with CH_2_Cl_2_ (3 × 10 mL). The organic layers were combined, washed with brine (10 mL), dried over MgSO_4_, and then dried in vacuo. The crude product was purified by column chromatography (SiO_2_, CH_2_Cl_2_ with a gradient of AcOEt (0 changing to 39:1 to 19:1 to 9:1 to 2:1). The product was isolated as a red solid (78.1 mg, 166 µmol, 37.8%). ^1^H NMR (500 MHz, CDCl_3_): *δ/*ppm 8.28 (s, 2H, H^A3^), 7.45 (d, *J* = 8.7 Hz, 4H, H^B2^), 7.25 (s, 2H, H^A5^), 6.68 (d, *J* = 8.5 Hz, 4H, H^B3^), 3.02 (s, 12H, H^NMe^), 2.65 (s, 6H, H^A6-Me^). ^13^C{^1^H} NMR (126 MHz, CDCl_3_): *δ*/ppm 158.0 (C^A6^), 150.7 (C^B4^), 133.4 (C^B2^), 124.6 (C^A5^), 120.2 (C^A3^), 111.9 (C^B3^), 109.0 (C^B1^), 95.3 (C^b^), 86.2 (C^a^), 40.3 (C^NMe^), 24.6 (C^A6-Me^); C^A2^, C^A4^ not resolved in HMBC. UV-VIS (CHCl_3_, 2.2 × 10^−5^ mol dm^−3^) λ/nm 303 (ε/dm^3^ mol^−1^ cm^−1^ 34,550), 358 (40,500), 439 (3,100). HR ESI-MS *m*/*z* 471.2548 [M + H]^+^ (calc. 471.2543). IR spectrum: see Figure S2 in the Supporting Information.

### 3.7. 4,4′-Bis(4-diphenylaminophenylethynyl)-6,6′-dimethyl-2,2′-bipyridine (***4***)

4,4′-Dibromo-6,6′-dimethyl-2,2′-bipyridine (100 mg, 292 µmol, 1.0 eq), Pd(dppf)Cl_2_ (12.8 mg, 17.5 µmol, 6 mol%), CuI (5.56 mg, 29.2 µmol, 10 mol%), 4-ethynyl-*N,N*-diphenylaniline (181 mg, 672 µmol, 2.3 eq) and PPh_3_ (7.66 mg, 29.2 µmol, 10 mol%) were loaded into a heat-dried microwave vial, previously degassed and purged with N_2_ three times. After 15 min of N_2_ purging, Et_3_N (583 µL) was added to the reaction mixture, which was then stirred at 80 °C overnight. After cooling to room temperature, the reaction mixture was poured into water (10 mL) and extracted with CH_2_Cl_2_ (3 × 10 mL). The organic layers were combined and washed with brine (10 mL). After back-extraction of the aqueous phase, the organic layers were dried over MgSO_4_ and then dried under reduced pressure. The product was recrystallized from a mixture of EtOH and CHCl_3_ and isolated as a dark orange powder (104 mg, 145 µmol, 49.5%). ^1^H NMR (500 MHz, CDCl_3_): *δ*/ppm 8.28 (s, 2H, H^A3^), 7.40 (d, *J* = 8.7 Hz, 4H, H^B2^), 7.29 (m, 4H, H^C3^), 7.25 (s, 2H, H^A5^), 7.13 (d, *J* = 7.5 Hz, 8H, H^C2^), 7.09 (t, *J* = 7.4 Hz, 4H, H^C4^), 7.01 (d, *J* = 8.9 Hz, 4H, H^B3^), 2.64 (s, 6H, H^Me^). ^13^C{^1^H} NMR (126 MHz, CDCl_3_): *δ*/ppm 158.0 (C^A6^), 148.9 (C^B4^), 147.0 (C^C1^), 132.9 (C^B2^), 129.5 (C^C3^), 125.2 (C^C2^), 124.8 (C^A5^), 123.9 (C^C4^), 121.8 (C^B3^), 114.8 (C^B1^), 94.0 (C^b^), 86.8 (C^a^), 24.7 (C^Me^); C^A2^, C^A4^ not resolved in HMBC. UV-VIS (CHCl_3_, 2.2 × 10^−5^ mol dm^−3^) λ/nm 302 (ε/dm^3^ mol^−1^ cm^−1^ 41,600), 380 (63,750), 468 (3300). HR EIS-MS *m*/*z* 719.3162 [M + H]^+^ (calc. 719.3169). IR spectrum: see Figure S4 in the Supporting Information.

### 3.8. [Cu(**1**)_2_][PF_6_]

Compound **1** (110 mg, 260 µmol, 2.0 eq) was dissolved in 100 mL of CH_2_Cl_2_/CH_3_CN (2:1, total volume 100 mL) in a round bottomed flask. After the addition of [Cu(MeCN)_4_][PF_6_] (48.5 mg, 130 µmol, 1.0 eq), the solution was stirred for 3 h at room temperature. Then, CH_2_Cl_2_ was removed in vacuo and the volume of CH_3_CN was reduced in vacuo. Then, Et_2_O was added to the reaction mixture to afford precipitation. The precipitate was collected by filtration, washed with Et_2_O and dried. [Cu(**1**)_2_][PF_6_] was isolated as a dark red powder (71.8 mg, 6.80 µmol, 52.4%). ^1^H NMR (500 MHz, CD_3_CN): *δ*/ppm 8.56 (br, 4H, H^A3^), 7.89 (d, *J* = 7.8 Hz, 8H, H^B2^), 7.75 (br, 4H, H^A5^), 6.89 (d, *J* = 8.1 Hz, 8H, H^B3^), 3.01 (s, 24H, H^NMe^), 2.33 (br, 12H, H^A6-Me^). ^13^C{^1^H} NMR (126 MHz, CD_3_CN): *δ*/ppm 153.1 (C^B4^), 150.6 (C^A4^), 129.0 (C^B2^), 124.4 (C^B1^), 122.2 (C^A5^), 116.4 (C^A3^), 113.3 (C^B3^), 40.4 (C^NMe^); C^A2^, C^A6^, C^A6-Me^ not resolved in HMBC. UV-VIS (CHCl_3_, 3.4 × 10^−6^ mol dm^−3^) λ/nm 313 (ε/dm^3^ mol^−1^ cm^−1^ 51,600), 370 (44,800), 500 sh (13,700). HRMS *m*/*z* 907.4228 [M − PF_6_]^+^ (calc. 907.4231), 423.2546, [**1** + H]^+^ (calc. 423.2543, base peak). IR spectrum: see Figure S22 in the Supporting Information.

### 3.9. [Cu(**2**)_2_][PF_6_]

The preparation of [Cu(**2**)_2_][PF_6_] was as for [Cu(**1**)_2_][PF_6_] starting with **2** (62.0 mg, 132 µmol, 1.0 eq) and [Cu(MeCN)_4_][PF_6_] (24.6 mg, 65.9 µmol, 0.5 eq). The product was isolated as a dark red solid (47.2 mg, 41 µmol, 36%). ^1^H NMR (500 MHz, acetone-*d*_6_): *δ*/ppm 8.61 (s, 4H, H^A3^), 7.68 (s, 4H, H^A5^), 7.51 (d, *J* = 8.8 Hz, 8H, H^B2^), 6.88 (broadened m, 8H, H^B3^), 3.09 (s, 24H, H^NMe^), 2.35 (br, 4H, H^A6-Me^). ^13^C{^1^H} NMR (126 MHz, acetone-*d*_6_): *δ*/ppm 158.4 (C^A6^), 152.7 (C^A2^), 152.2 (C^B4^), 135.4 (C^A4^), 134.3 (C^B2^), 127.9 (C^A5^), 122.3 (C^A3^), 113.2 (C^B3^), 109.6 (C^B1^), 99.5 (C^b^), 86.7 (C^a^), 40.5 (C^NMe^), 25.2 (C^A6-Me^). UV-VIS (CHCl_3_, 1.0 × 10^−5^ mol dm^−3^) λ/nm 263 (ε/dm^3^ mol^−1^ cm^−1^ 39,400), 326 (40,700), 409 (41,800), 515 (15,200). ESI-MS *m*/*z* 1003.4226 [M − PF_6_]^+^ (calc. 1003.4231), 471.2545 [**2** + H]^+^ (calc. 471.2543, base peak). IR spectrum: see Figure S23 in the Supporting Information.

### 3.10. [Cu(**3**)_2_][PF_6_]

[Cu(**3**)_2_][PF_6_] was prepared in the same manner as [Cu(**1**)_2_][PF_6_] starting with **3** (88.2 mg, 131 µmol, 2.0 eq) and [Cu(MeCN)_4_][PF_6_] (24.5 mg, 65.7 µmol, 1.0 eq). [Cu(**3**)_2_][PF_6_] was isolated as a dark red powder (34.0 mg, 22.0 µmol, 33.1%). ^1^H NMR (500 MHz, CD_3_CN): *δ*/ppm 8.57 (s, 4H, H^A3^), 7.84 (d, *J* = 8.5 Hz, 8H, H^B2^), 7.75 (s, 4H, H^A5^), 7.36 (t, *J* = 7.9 Hz, 16H, H^C3^), 7.17–7.12 (m, 24H, H^C2+C4^), 7.11 (d, *J* = 8.7 Hz, 8H, H^B3^), 2.32 (s, 12H, H^Me^). ^13^C{^1^H} NMR (126 MHz, CD_3_CN): *δ*/ppm 149.8 (C^B4^), 147.8 (C^C1^), 130.7 (C^C3^), 129.5 (C^B1^), 129.2 (C^B2^), 126.2 (C^C2/C4^), 125.3 (C ^C2/C4^), 123.3 (C^A5^), 122.9 (C^B3^), 117.6 (C^A3^), 25.4 (C^A6-Me^); C^A2^, C^A4^, C^A6^, C^B4^ not resolved in HMBC. UV-VIS (CHCl_3_, 1.0 × 10^−5^ mol dm^−3^) λ/nm 309 (ε/dm^3^ mol^−1^ cm^−1^ 67,050), 319 sh (63,400), 394 (56,000), 493 (19,100). HRMS *m/z* 1405.5492 [M − PF_6_]^+^ (calc. 1404.5551, base peak, see Figure S19). IR spectrum: see Figure S24 in the Supporting Information.

### 3.11. [Cu(**4**)_2_][PF_6_]

[Cu(**4**)_2_][PF_6_] was prepared following the methods described for [Cu(**1**)_2_][PF_6_] starting with **4** (83.2 mg, 116 µmol, 2.0 eq) and [Cu(MeCN)_4_][PF_6_] (21.6 mg, 57.9 µmol, 1.0 eq). [Cu(**4**)_2_][PF_6_] was isolated as a dark red powder (40.0 mg, 24.0 µmol, 40.0%). ^1^H NMR (500 MHz, CD_3_CN): *δ*/ppm 8.33 (s, 4H, H^A3^), 7.55 (s, 4H, H^A5^), 7.45 (d, *J* = 8.7 Hz, 8H, H^B2^), 7.36 (t, *J* = 7.9 Hz, 16H, H^C3^), 7.19–7.12 (overlapping m, 24H, H^C2+C4^), 6.97 (d, *J* = 8.7 Hz, 8H, H^B3^), 2.28 (s, 12H, H^Me^). ^13^C{^1^H} NMR (126 MHz, CD_3_CN): *δ*/ppm 150.1 (C^B4^), 147.2 (C^C1^), 134.0 (C^B2^), 130.7 (C^C3^), 127.9 (C^A5^), 126.7 (C^C2^), 125.6 (C^C4^), 122.2 (C^A3^), 121.8 (C^B3^), 113.7 (C^B1^), 97.3 (C^b^), 86.4 (C^a^), 25.5 (C^Me^); C^A2^, C^A4^, C^A6^ not resolved in HMBC. UV-VIS (CHCl_3_, 1.0 × 10^−5^ mol dm^−3^) λ/nm 298 (ε/dm^3^ mol^−1^ cm^−1^ 88,440), 331 (72,250), 422 (83,500), 507 (33,000). HRMS *m*/*z* 1500.5499 [M − PF_6_]^+^ (calc. 1500.5517, base peak). IR spectrum: see Figure S25 in the Supporting Information.

### 3.12. Calculations

Ground state density functional theory DFT calculations were carried out using Spartan ‘18 (v. 1.3) [74] at the B3LYP level with a 6-31G* basis set in vacuum. We have previously demonstrated that for bis(diimine)copper(I) complexes, the choice of atomic orbital basis set (6-311++G** basis set on all atoms, 6-311++G** on Cu and 6-31G* basis set on C, H and N, or 6-31G* basis set on all atoms) has a negligible effect on the calculated MO compositions, while significantly influencing the calculated absorption spectra [75]. Hence, a 6-31G* basis set on all atoms was chosen to optimize computer time. Geometry optimization was also carried out at the DFT level after an initial geometry energy optimization had been completed at a semi-empirical (PM3) level. For the complex [Cu(**2**)_2_]^+^, a restricted hybrid HF-DFT SCF calculation was also performed using Pulay DIIS plus Geometric Direct Minimization Polarizable Continuum solvation model in CHCl_3_ using Spartan ‘18 (v. 1.3) [74].

### 3.13. DSC Fabrication

FTO/TiO_2_ electrodes (Solaronix Test Cell Titania Electrodes, Solaronix SA, Aubonne, Switzerland) were washed with milliQ water and EtOH, heated at 450 °C for 30 min, and then cooled to ca. 80 °C. The electrodes were immediately immersed in a DMSO solution of **5** (1.0 mM) for 24 h, after which they were removed, washed with DMSO and EtOH, and finally dried in a stream of N_2_. To assemble the adsorbed heteroleptic dyes, each functionalized electrode was placed in a CH_2_Cl_2_ solution (0.1 mM) of [CuL_2_][PF_6_] (L = **1**, **2**, **3** or **4**) for 3 days at room temperature, and was then removed, washed with CH_2_Cl_2_ and dried under an N_2_ stream. For the reference dye N719 (Solaronix SA, Aubonne, Switzerland), FTO/TiO_2_ electrodes (Solaronix Test Cell Titania Electrodes, Solaronix SA, Aubonne, Switzerland) were immersed in a solution of N719 (EtOH, 0.3 mM) for 1 day. Then the electrodes were removed from the solution, washed with EtOH and dried in a stream of N_2_. Counter electrodes (Solaronix Test Cell Platinum Electrodes, Solaronix SA, Aubonne, Switzerland) were washed with EtOH and then heated at 450 °C for 30 min to remove volatile organic impurities.

The working and counter-electrode for each DSC were joined together using thermoplast hot-melt sealing foil (Solaronix Test Cell Gaskets, 60 µm, Solaronix SA, Aubonne, Switzerland) and the gap between them was filled with electrolyte (LiI (0.1 M), I_2_ (0.05 M), 1-methylbenzimidazole (0.5 M), 1-butyl-3-methylimidazolinium iodide (0.6 M) in 3-methoxypropionitrile) by vacuum backfilling through a hole in the counter-electrode. Finally, the hole was sealed (Solaronix Test Cell Sealings and Solaronix Test Cell Caps, Solaronix SA, Aubonne, Switzerland).

### 3.14. Electrodes for Solid-State Absorption Spectroscopy

The method in Section 3.13 was used to fabricate dye-functionalized electrodes, but starting with Solaronix Test Cell Titania Electrodes Transparent (Solaronix SA, Aubonne, Switzerland).

### 3.15. DSCs with Co-Adsorbent n-Decylphosphonic Acid

The method in Section 3.13 was used to fabricate dye-functionalized electrodes, but with one change to the dye bath procedure. In place of a DMSO solution of **5** (1.0 mM), a DMSO solution of containing both **5** (1.0 mM) and *n*-decylphosphonic acid (1.0 mM) was used. The dipping time was 24 h.

### 3.16. DSC, EQE and EIS Measurements

The DSCs were all masked before measurements. The mask was made from a black-coloured copper sheet with an accurately calibrated aperture smaller than the surface area of TiO_2_. Cells were also masked on the top and on the sides using black tape. Performance measurements were made by irradiating the DSC from behind with a LOT Quantum Design LS0811 instrument (LOT-QuantumDesign GmbH, Darmstadt, Germany, 100 mW cm^−2^ = 1 sun, AM1.5 G conditions) and the simulated light power was calibrated with a silicon reference cell.

EQE measurements used a Spe Quest quantum efficiency setup (ReRa Systems, Nijmegen, The Netherlands) with a 100 W halogen lamp (QTH) and a lambda 300 grating monochromator (LOT-Oriel GmbH & Co. KG, Darmstadt, Germany). The monochromatic light was modulated to 3 Hz using a chopper wheel (ThorLabs Inc., Newton, NJ, USA), and the cell response was amplified with a large dynamic range IV converter (Melles Griot B.V., Didam, the Netherlands) and measured with a SR830 DSP Lock-In amplifier (Stanford Research Systems Inc., Sunnyvale, CA, USA).

EIS measurements were performed using a ModuLab^®^ XM PhotoEchem photoelectrochemical measurement system (Solartron Metrology Ltd., Leicester, UK). The impedance was measured in galvanostatic mode at open-circuit potential of the cell at a light intensity of 22 mW cm^−2^ (590 nm) in the frequency range 0.05 Hz to 400 kHz using an amplitude of 10 mV. The impedance data were analysed using ZView^®^ software (Scribner Associates Inc., Southern Pines, NC, USA).

## 4. Conclusions

We have described the syntheses and characterisations of ligands **1**–**4** and the homoleptic copper(I) complexes [Cu(**1**)_2_][PF_6_], [Cu(**2**)_2_][PF_6_], [Cu(**3**)_2_][PF_6_] and [Cu(**4**)_2_][PF_6_]. The ligands feature electron-donating Me_2_N or Ph_2_N groups, and compounds **2** and **4** contain alkynyl spacers. A comparison of the solution absorption spectra of **2** with **1**, and of **4** with **3** confirms that increasing the π-conjugation as the alkynyl units are introduced extends the absorption towards longer wavelengths. The solution absorption spectra of the complexes exhibit absorptions in the visible region assigned to a combination of ILCT and MLCT. DSCs containing heteroleptic [Cu(**5**)(L_ancillary_)]^+^ dyes with L_ancillary_ = **1**–**4**, and a phosphonic acid anchoring ligand **5** were fabricated. The best performing DSCs were sensitized with [Cu(**5**)(**1**)]^+^ and [Cu(**5**)(**3**)]^+^. The presence of the alkynyl spacers in the dyes [Cu(**5**)(**2**)]^+^ and [Cu(**5**)(**4**)]^+^ results in lower values of *J*_SC_ and EQE_max_ than for [Cu(**5**)(**1**)]^+^ and [Cu(**5**)(**3**)]^+^. Addition of a co-absorbent (*n*-decylphosphonic acid) to [Cu(**5**)(**1**)]^+^ in the DSCs did not significantly improve performance. Using EIS, we have demonstrated a more favourable electron injection into TiO_2_ when the dye is [Cu(**5**)(**1**)]^+^, and have confirmed that the introduction of the alkynyl spacers is not beneficial.

## Figures and Tables

**Table 1:** Performance parameters (under 1 sun illumination) of duplicate, masked DSCs on the day of sealing the cells with the dyes [Cu(**5**)(**1**)]^+^ and [Cu(**5**)(**2**)]^+^. The data are compared to a DSC containing N719 and relative *η* values are with respect to N719 set at 100%.

Dye	Cell Number	*J*_SC_/mA cm^−2^	*V*_OC_/mV	*ff*/%^1^	*η/*%	Relative *η*/%
[Cu(**5**)(**1**)]^+^	1	4.54	541	67.6	1.66	31.0
[Cu(**5**)(**1**)]^+^	2	4.69	539	68.1	1.72	32.1
[Cu(**5**)(**1**)]^+^	3	4.74	539	70.1	1.79	33.4
[Cu(**5**)(**1**)]^+^	4	4.55	524	69.7	1.66	31.0
[Cu(**5**)(**2**)]^+^	1	3.59	514	70.7	1.30	24.3
[Cu(**5**)(**2**)]^+^	2	3.64	508	69.6	1.29	24.0
[Cu(**5**)(**2**)]^+^	3	3.54	513	71.2	1.29	24.1
[Cu(**5**)(**2**)]^+^	4	3.57	514	68.4	1.25	23.4
N719		14.13	602	63.0	5.36	100

^1^*ff* = fill-factor.

**Table 2:** Performance parameters (under 1 sun illumination) of duplicate, masked DSCs on the day of sealing the cells with the dyes [Cu(**5**)(**3**)]^+^ and [Cu(**5**)(**4**)]^+^. The data are compared to a DSC containing N719 and relative *η* values are with respect to N719 set at 100%.

Dye	Cell Number	*J*_SC_/mA cm^−2^	*V*_OC_/mV	*ff*/%	*η/*%	Relative *η*/%
[Cu(**5**)(**3**)]^+^	1	4.82	532	67.9	1.74	32.5
[Cu(**5**)(**3**)]^+^	2	5.25	523	70.3	1.93	36.0
[Cu(**5**)(**3**)]^+^	3	4.64	536	68.6	1.71	31.8
[Cu(**5**)(**3**)]^+^	4	4.89	533	66.8	1.74	32.4
[Cu(**5**)(**4**)]^+^	1	4.24	535	69.3	1.57	29.3
[Cu(**5**)(**4**)]^+^	2	3.96	530	68.6	1.44	26.8
[Cu(**5**)(**4**)]^+^	3	4.08	534	66.8	1.46	27.2
[Cu(**5**)(**4**)]^+^	4	4.05	531	70.3	1.51	28.2
N719		14.25	603	60.3	5.18	100

**Table 3:** Performance parameters (under 1 sun illumination) of duplicate, masked DSCs on the day of sealing the cells the dye [Cu(**5**)(**1**)]^+^ and co-adsorbent DPA. The data are compared to a DSC containing N719 and relative *η* values are with respect to N719 set at 100%.

Dye	Cell Number	*J*_SC_/mA cm^−2^	*V*_OC_/mV	*ff*/%	*η/*%	Relative *η*/%
[Cu(**5**)(**1**)]^+^	1	4.75	534	65.1	1.65	30.8
[Cu(**5**)(**1**)]^+^	2	4.75	548	68.9	1.79	33.4
[Cu(**5**)(**1**)]^+^	3	4.42	545	65.9	1.59	29.6
[Cu(**5**)(**1**)]^+^	4	4.73	543	68.8	1.77	32.9
N719		14.13	602	63.0	5.36	100

**Table 4:** EIS parameters^1^ obtained from fitting the experimental data for DSCs with dyes [Cu(**5**)(**1**)]^+^ and [Cu(**5**)(**2**)]^+^, and with [Cu(**5**)(**1**)]^+^ with the co-adsorbent *n*-decylphosphonic acid (DPA). Data are for the most representative DSC of a set of four; data for all the DSCs are given in Table S1 (see Supporting Information).

Dye	R_rec_/Ω	C_μ_/μF	R_tr_/Ω	τ/ms	τ_t_/ms	Ld/μm	R_s_/Ω	R_Pt_/Ω	C_Pt_/μF	*J*_SC_/mA cm^−2^	*V*_OC_/mV	*ƞ*/%
[Cu(**5**)(**1**)]^+^	125	386	28	48	11	25	20	24	5	4.69	539	1.72
[Cu(**5**)(**2**)]^+^	200	328	30	66	10	31	10	16	6	3.54	513	1.29
[Cu(**5**)(**1**)]^+^ + DPA	384	252	39	97	10	38	10	21	6	4.75	548	1.79

^1^ R_rec_ = recombination resistance; C_µ_ = chemical capacitance; R_tr_ = transport resistance; τ = lifetime’ L_d_ = electron diffusion length; R_s_ = series resistance; R_Pt_ and C_Pt_ = Pt counter electrode resistance and capacitance.

## Supplementary Materials

The following are available online: Figures S1–S4: IR spectra of compounds **1**–**4**; Figures S5–S16: NMR spectra of compounds **1**–**4**; Figures S17–S20: high-resolution electrospray mass spectra of [CuL_2_][PF_6_] with L = **1**–**4**; Figures S21–S24: IR spectra of [CuL_2_][PF_6_] with L = **1**–**4**; Figures S25–S36: NMR spectra of [CuL_2_][PF_6_] with L = **1**–**4**; Figure S37: orbital compositions of HOMOs and LUMOs in [Cu(**2**)_2_]^+^ using a polarizable continuum solvation model; Figure S38: orbital compositions (DFT) of the highest occupied and lowest unoccupied MOs in [Cu(**5**)(**1**)]^+^; Figures S39 and S40: *J–V* curves and EQE spectra for DSCs with [Cu(**5**)(**1**)]^+^ and [Cu(**5**)(**2**)]^+^ compared to N719; Table S1. EIS parameters for all DSCs.
