# Supplementary material for: Are Alkynyl Spacers in Ancillary Ligands in Heteroleptic Bis(diimine)copper(I) Dyes Beneficial for Dye Performance in Dye-Sensitized Solar Cells?
Source: Molecules. 2020 Mar 27;25(7):1528. doi: 10.3390/molecules25071528 (PMC7180879; doi:10.3390/molecules25071528)
Supplement: Supplementary file 1 [file molecules-25-01528-s001.pdf]

Supporting Information

Are alkynyl spacers in ancillary ligands in heteroleptic bis(diimine)copper(I) dyes beneficial for dye performance in dye-sensitized solar cells?

Guglielmo Risi,[1] **Mariia Becker**,[1] **Catherine E. Housecroft** [1], **and Edwin C. Constable** [1*]

[1]Department of Chemistry, University of Basel, BPR 1096, Mattenstrasse 24a, CH-4058 Basel, Switzerland; guglielmo.risi@unibas.ch (G.R.); mariia.karpacheva@unibas.ch (M.B.); catherine.housecroft@unibas.ch (C.E.H.); edwin.constable@unibas.ch (E.C.C.)

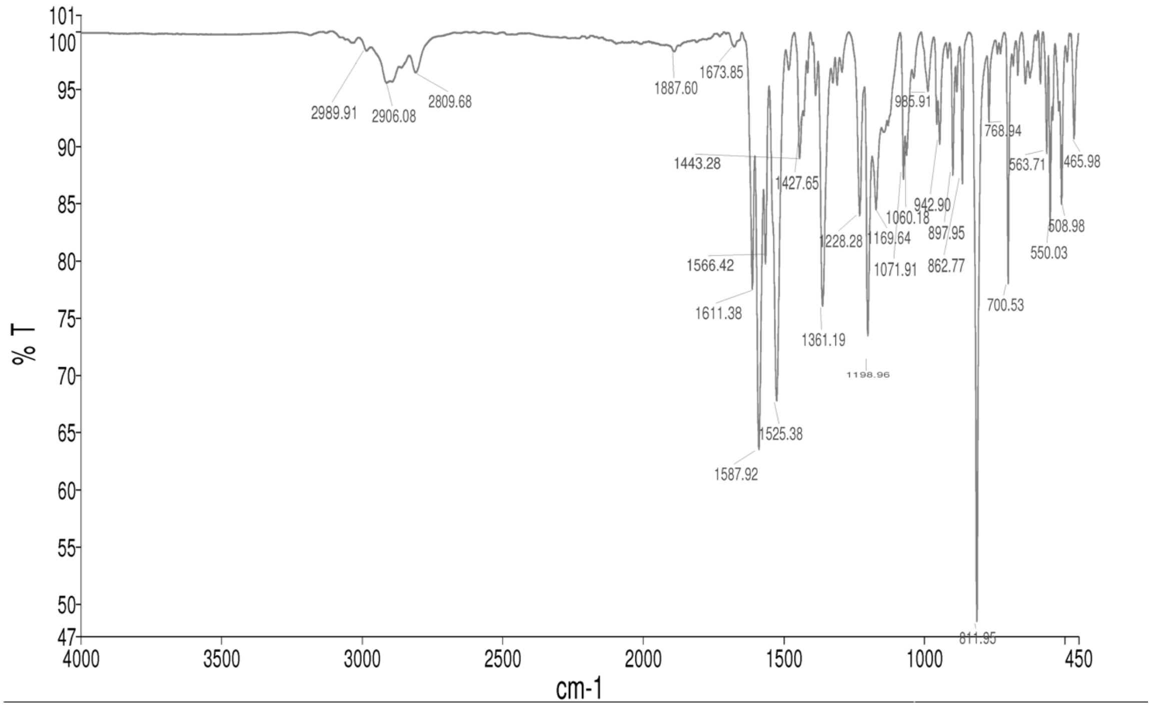

Figure S1. FT-IR spectrum of **1** (solid state).

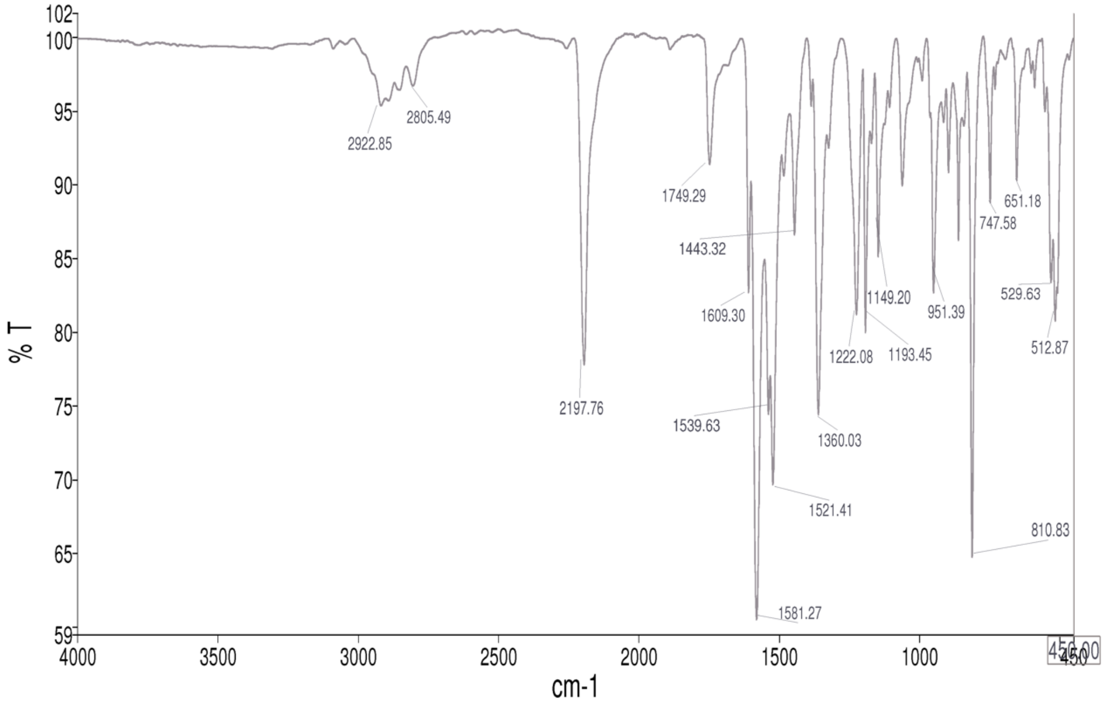

Figure S2. FT-IR spectrum of **2** (solid state).

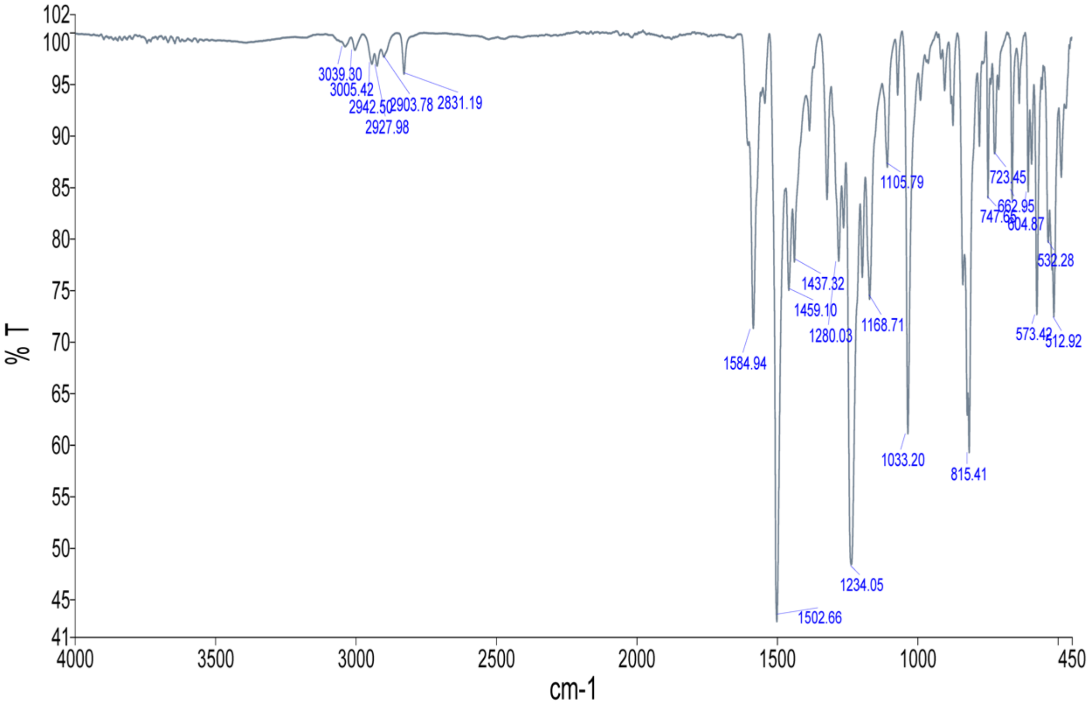

Figure S3. FT-IR spectrum of **3** (solid state).

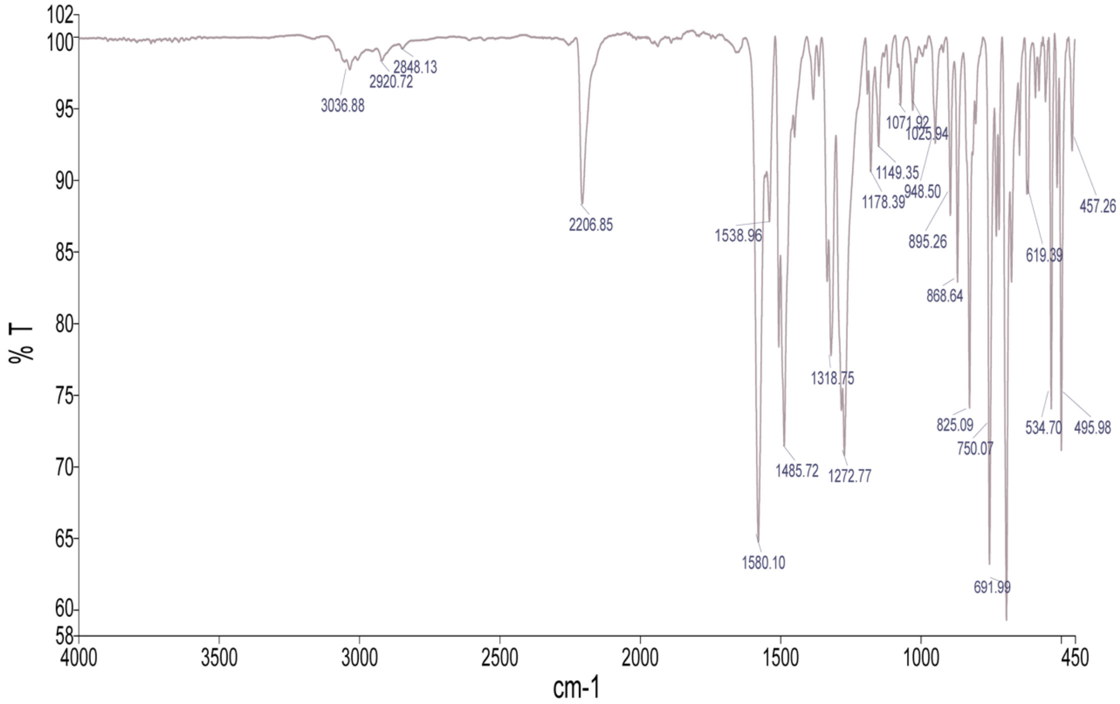

Figure S4. FT-IR spectrum of **4** (solid state).

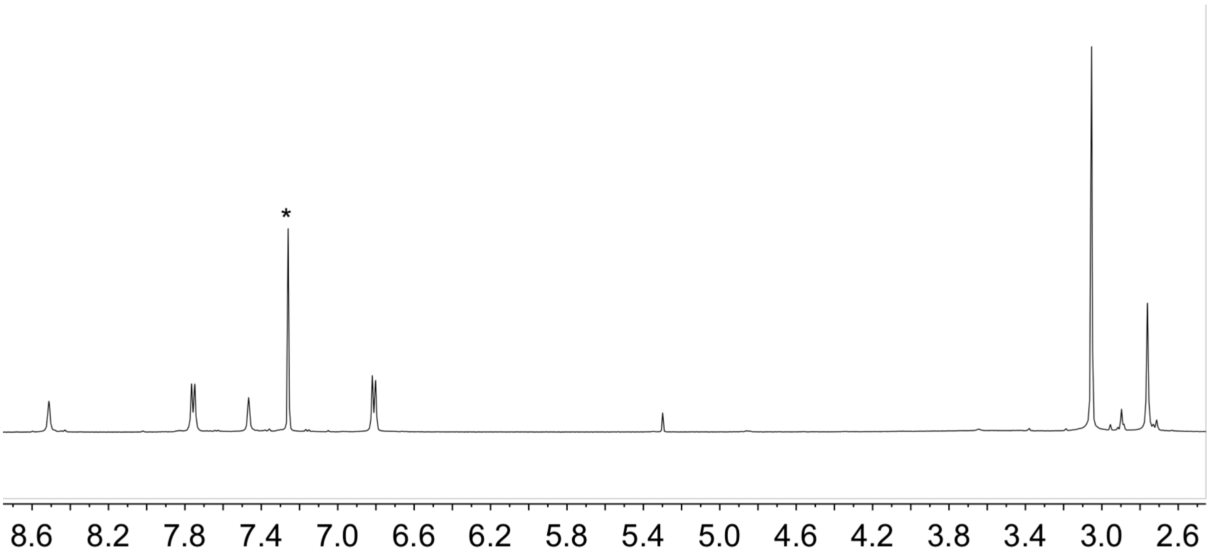

Figure S5. ^{1}H NMR spectrum (500 MHz, $CDCl_3$, 298 K) of compound **1**. * = residual $CHCl_3$. Scale: δ / ppm.

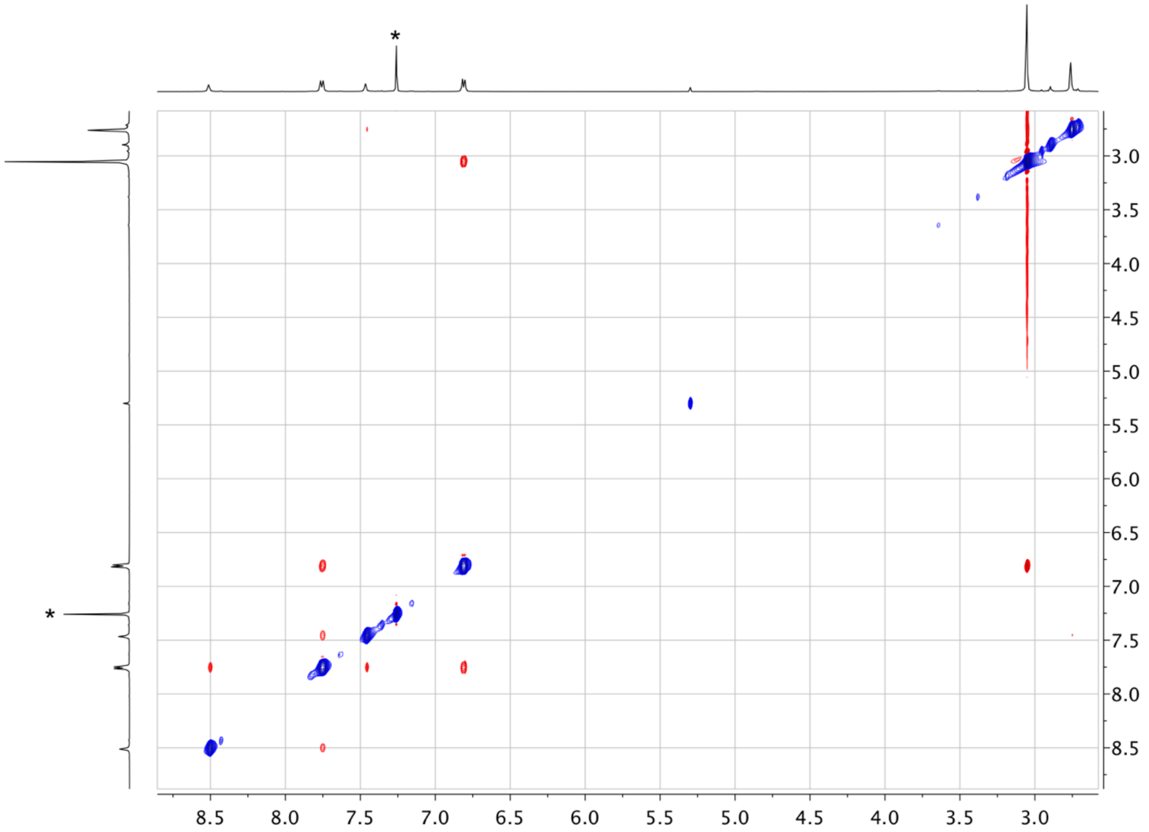

Figure S6. NOESY spectrum (500 MHz, $CDCl_3$, 298 K) of compound **1**. * = residual $CHCl_3$. Scale: δ / ppm.

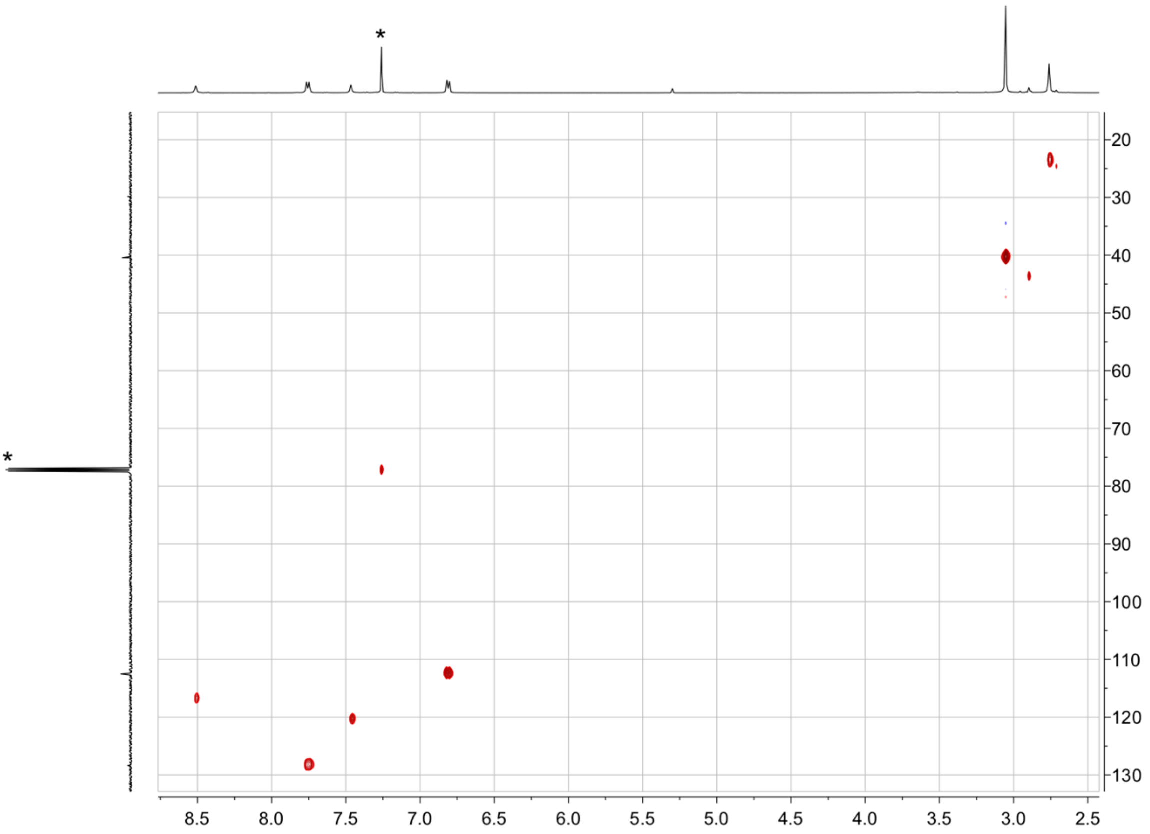

Figure S7. HMQC spectrum (500 MHz ^{1}H, 126 MHz ^{13}C, $CDCl_3$, 298 K) of compound **1**. * = $CDCl_3$ or residual $CHCl_3$. Scale: δ / ppm.

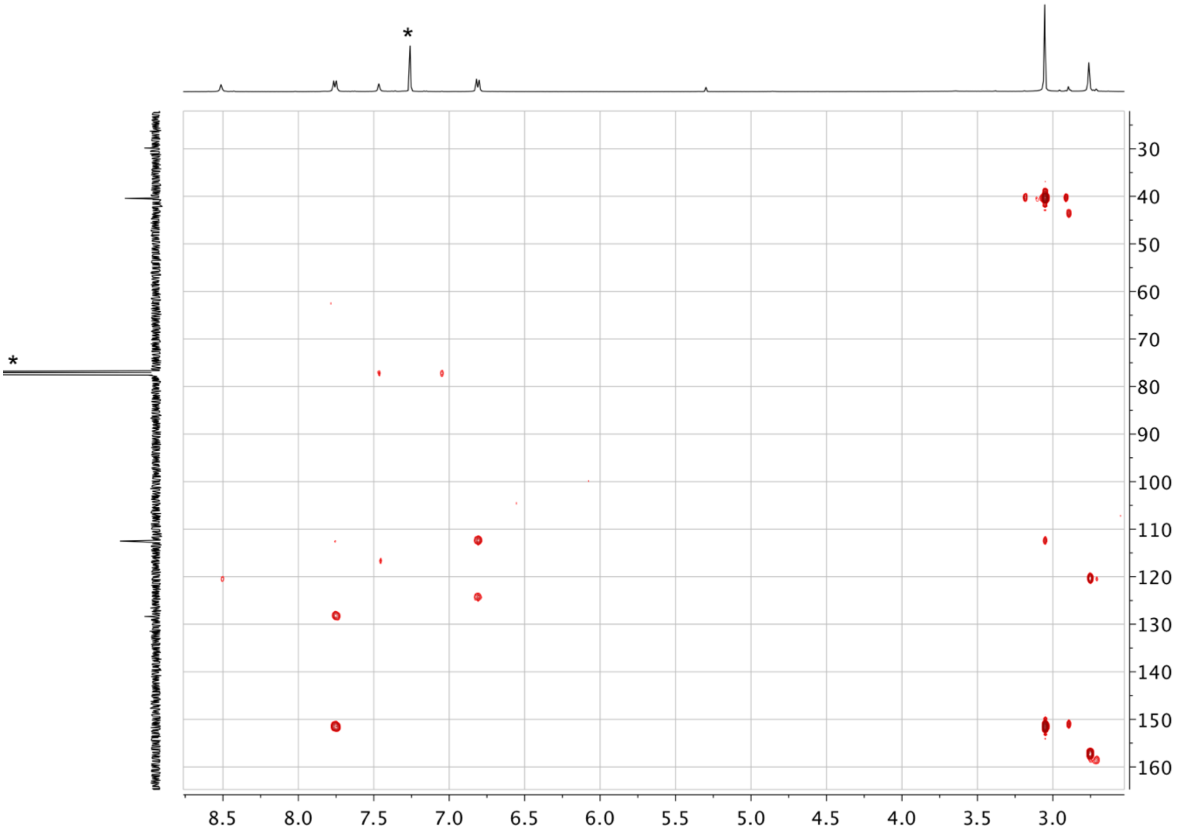

Figure S8. HMBC spectrum (500 MHz ^{1}H, 126 MHz ^{13}C, $CDCl_3$, 298 K) of compound **1**. * = $CDCl_3$ or residual $CHCl_3$. Scale: δ / ppm.

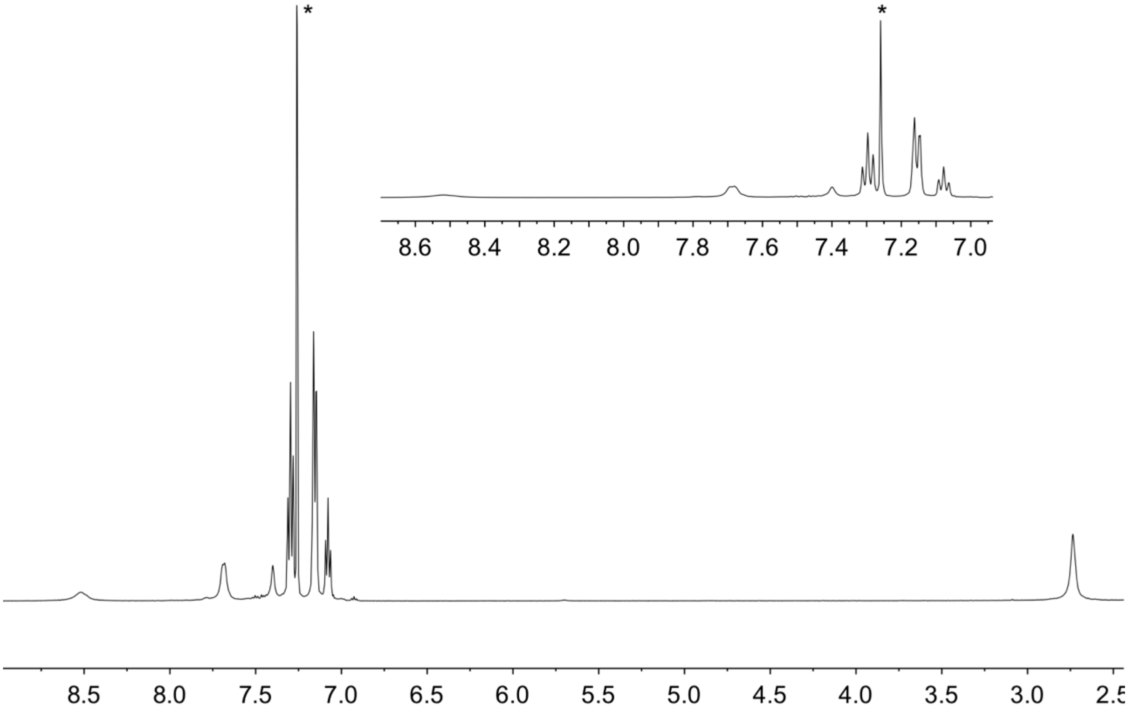

Figure S9. ^1H NMR spectrum (500 MHz, $CDCl_3$, 298 K) of compound **3** with inset of the aromatic region. * = residual $CHCl_3$. Scale: δ / ppm.

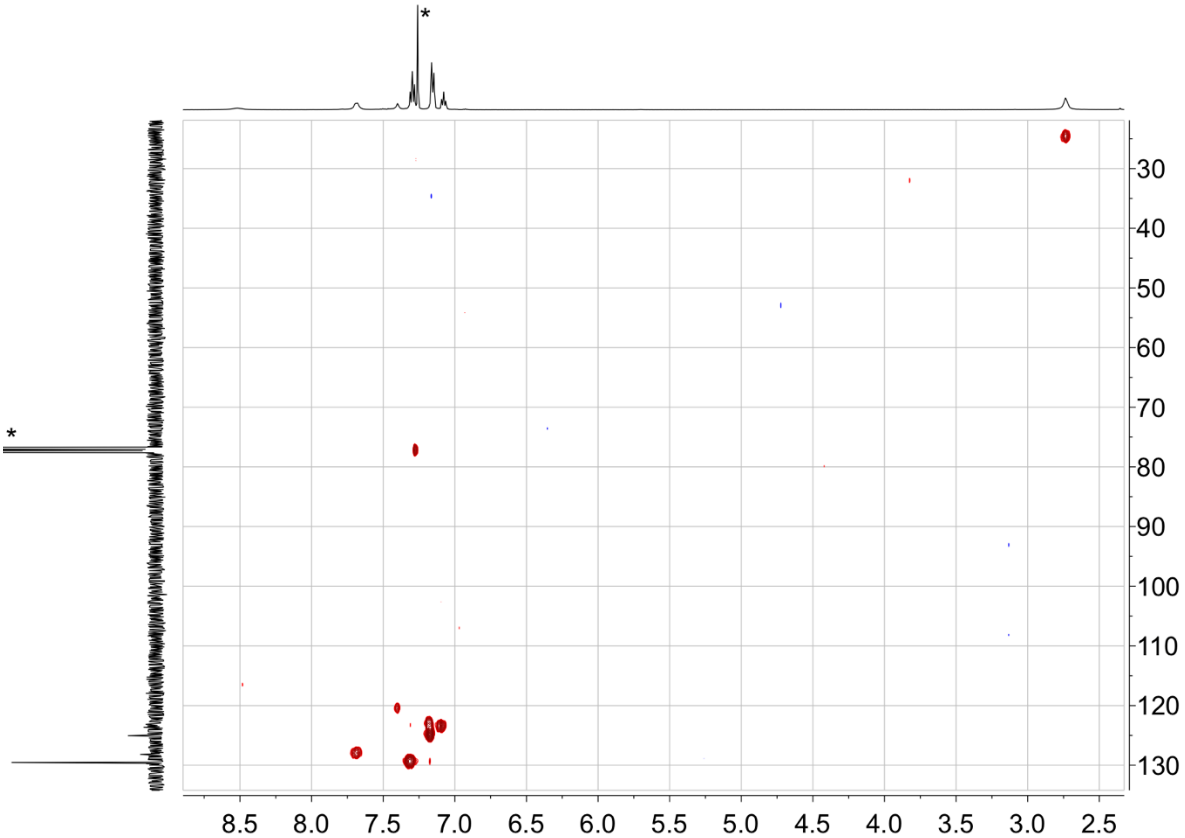

Figure S10. HMQC spectrum (500 MHz ^1H, 126 MHz ^{13}C, $CDCl_3$, 298 K) of compound **3**. * = $CDCl_3$ or residual $CHCl_3$. Scale: δ / ppm.

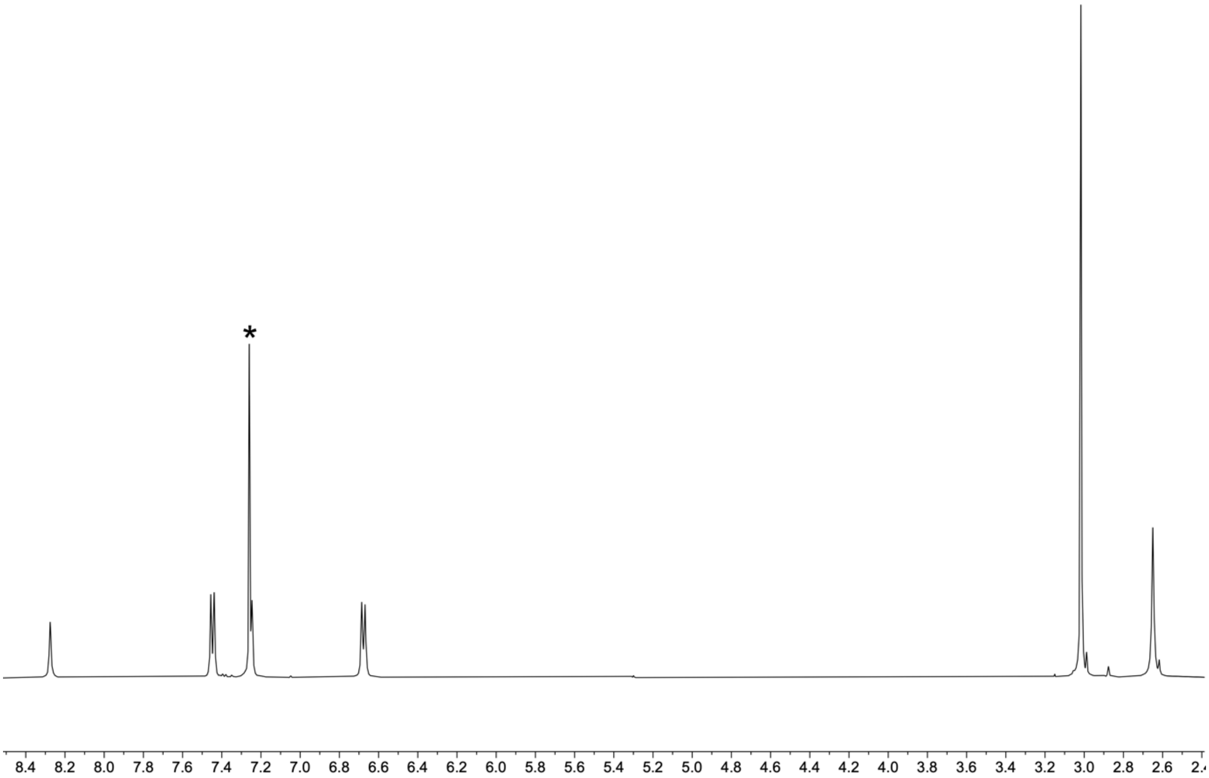

Figure S11. ^{1}H NMR spectrum (500 MHz, $CDCl_3$, 298 K) of compound **2**. * = residual $CHCl_3$. Scale: δ / ppm.

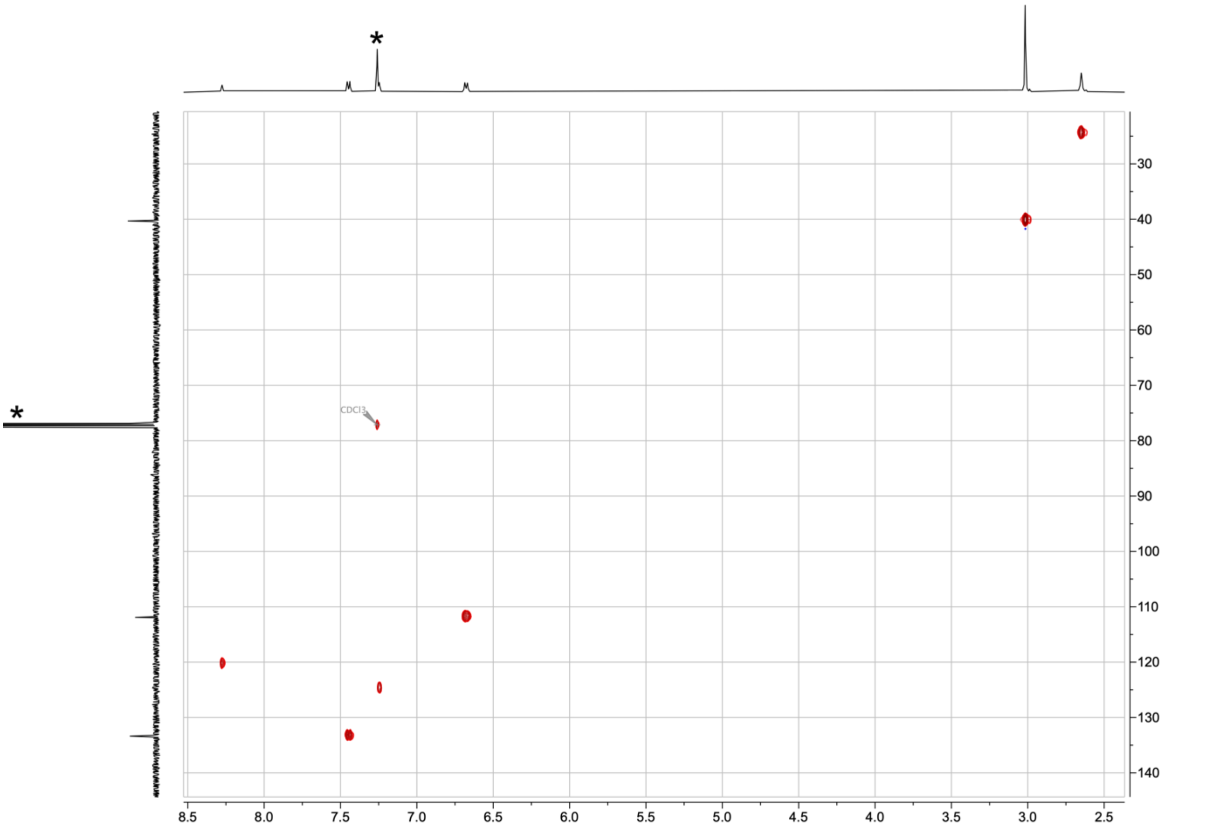

Figure S12. HMQC spectrum (500 MHz ^{1}H, 126 MHz ^{13}C, $CDCl_3$, 298 K) of compound **2**. * = $CDCl_3$ or residual $CHCl_3$. Scale: δ / ppm.

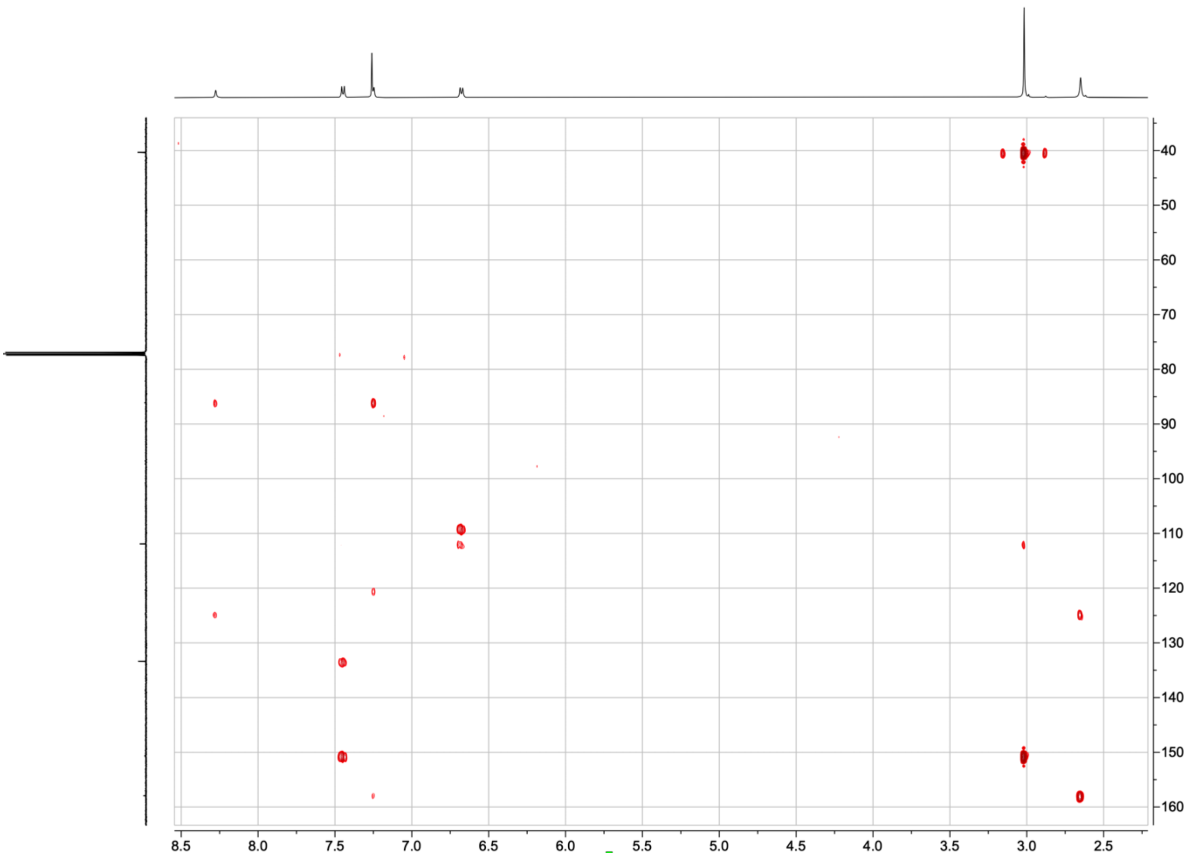

Figure S13. HMBC spectrum (500 MHz ^{1}H, 126 MHz ^{13}C, $CDCl_3$, 298 K) of compound **2**. * = $CDCl_3$ or residual $CHCl_3$. Scale: δ / ppm.

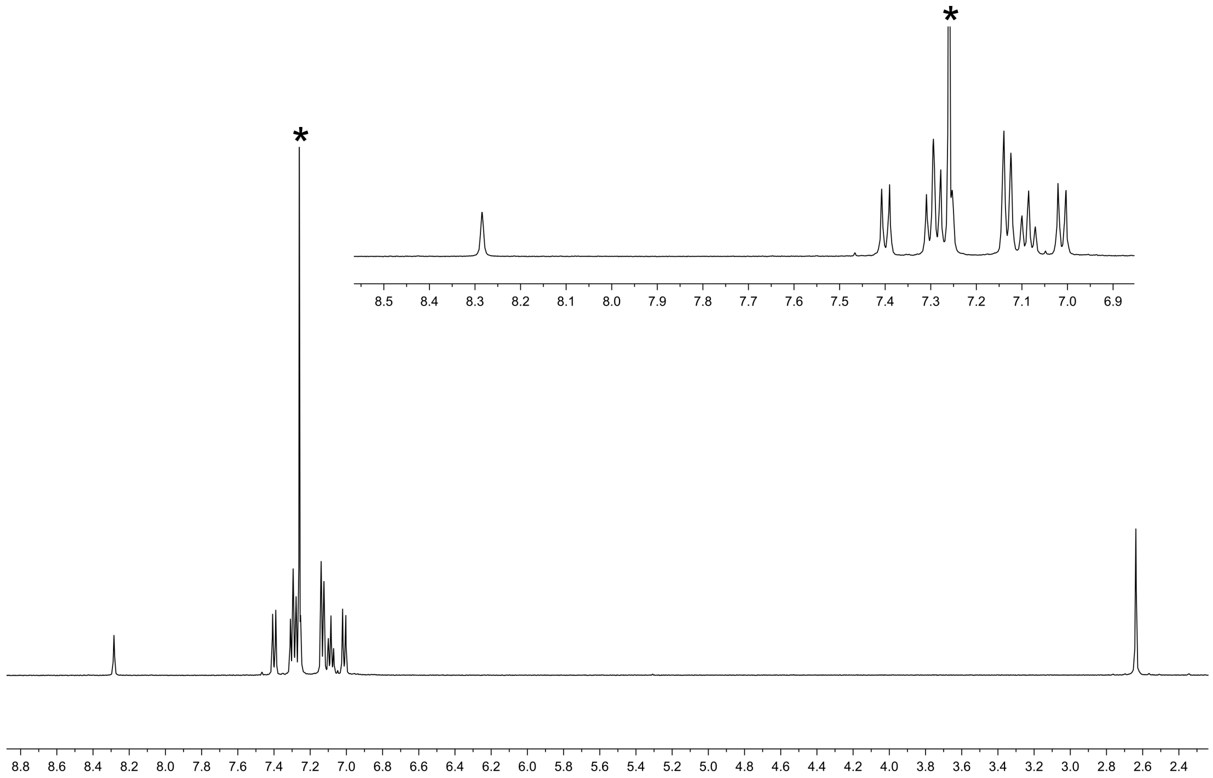

Figure S14. ^{1}H NMR spectrum (500 MHz, $CDCl_3$, 298 K) of compound **4** and inset, expansion of the aromatic region. * = residual $CHCl_3$. Scale: δ / ppm.

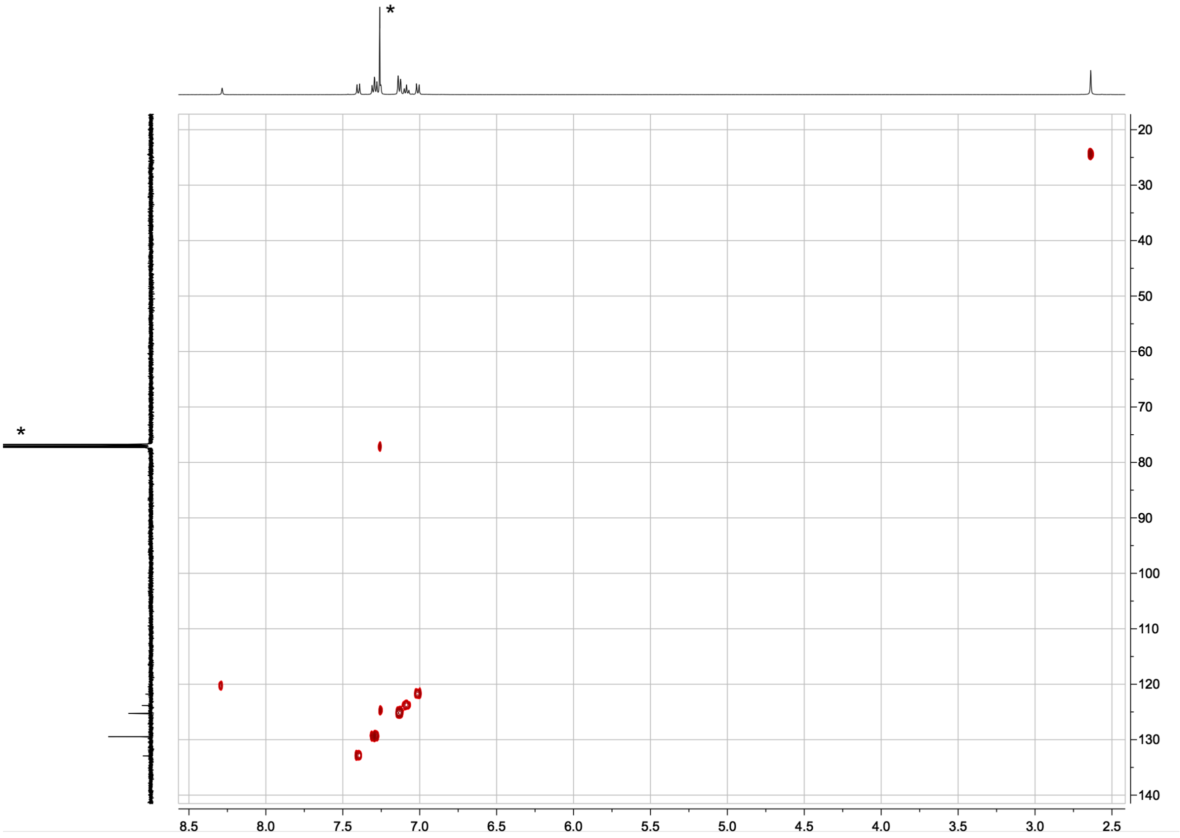

Figure S15. HMQC spectrum (500 MHz ^{1}H, 126 MHz ^{13}C, $CDCl_3$, 298 K) of compound **4**. * = $CDCl_3$ or residual $CHCl_3$. Scale: δ / ppm.

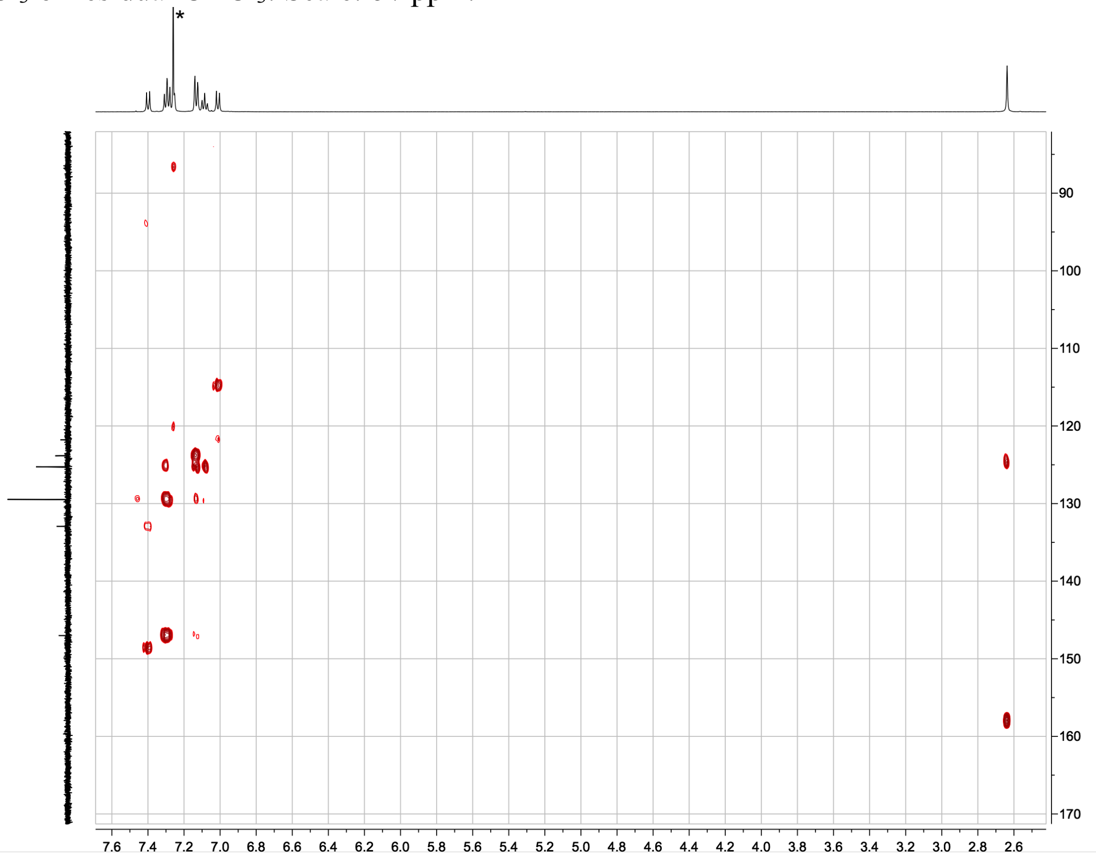

Figure S16. HMBC spectrum (500 MHz ^{1}H, 126 MHz ^{13}C, $CDCl_3$, 298 K) of compound **4**. * = $CDCl_3$ or residual $CHCl_3$. Scale: δ / ppm.

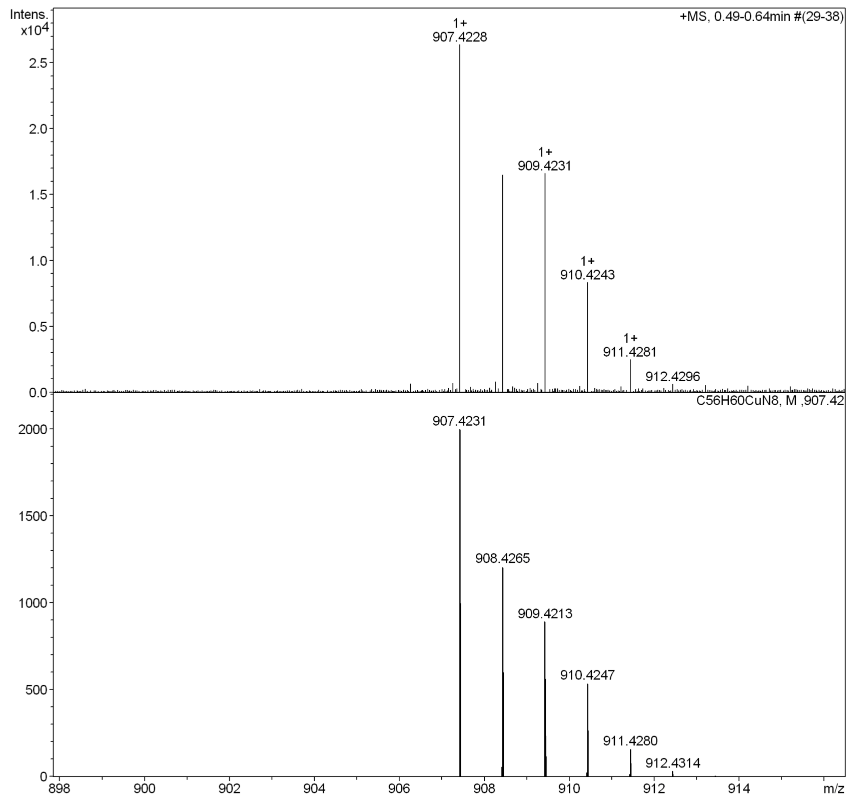

Figure S17. High-resolution electrospray mass spectrum of [Cu(**1**)$_2$][PF$_6$] showing the [M–PF$_6$]$^+$ ion and predicted isotope pattern.

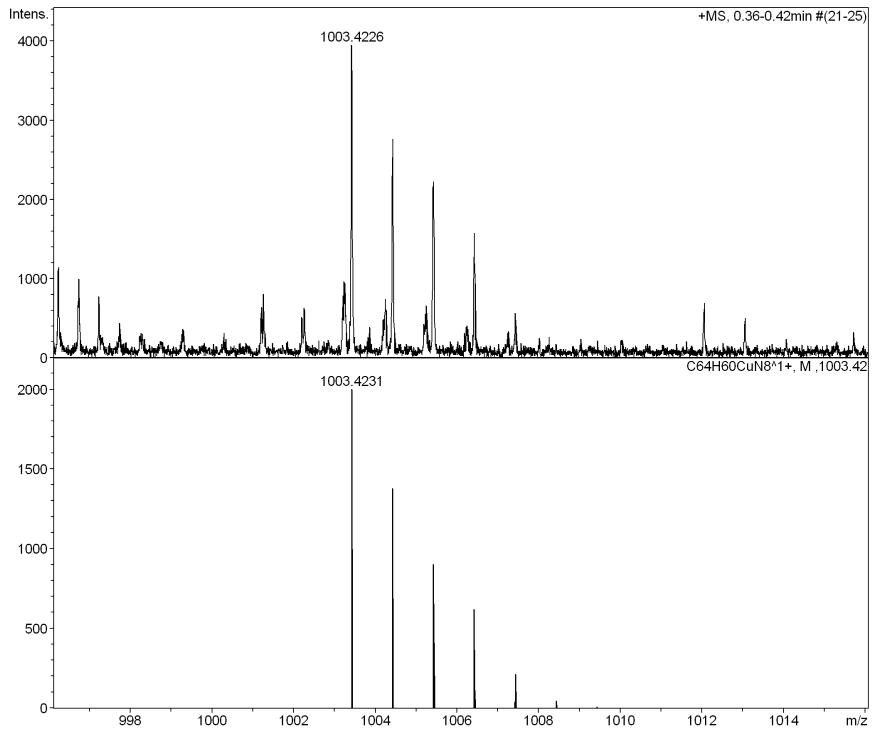

Figure S18. High-resolution electrospray mass spectrum of [Cu(**2**)$_2$][PF$_6$] showing the [M–PF$_6$]$^+$ ion and predicted isotope pattern.

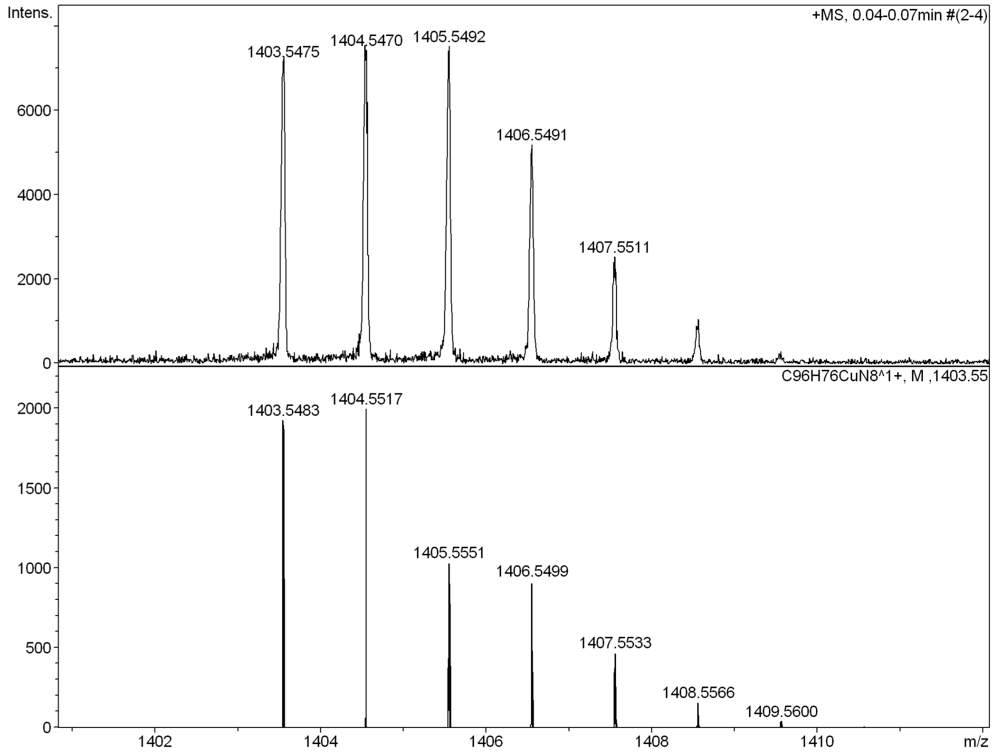

Figure S19. High-resolution electrospray mass spectrum of [Cu(**3**)$_2$][PF_6] showing the [M–PF_6]$^+$ ion and predicted isotope pattern.

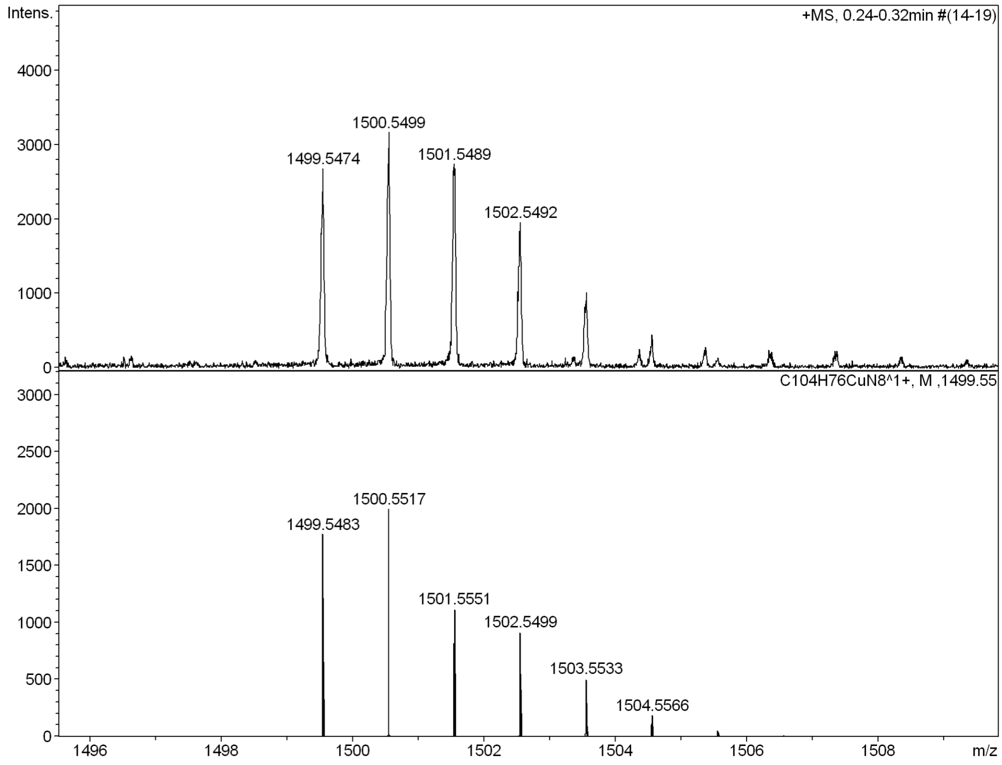

Figure S20. High-resolution electrospray mass spectrum of [Cu(**4**)$_2$][PF_6] showing the [M–PF_6]$^+$ ion and predicted isotope pattern.

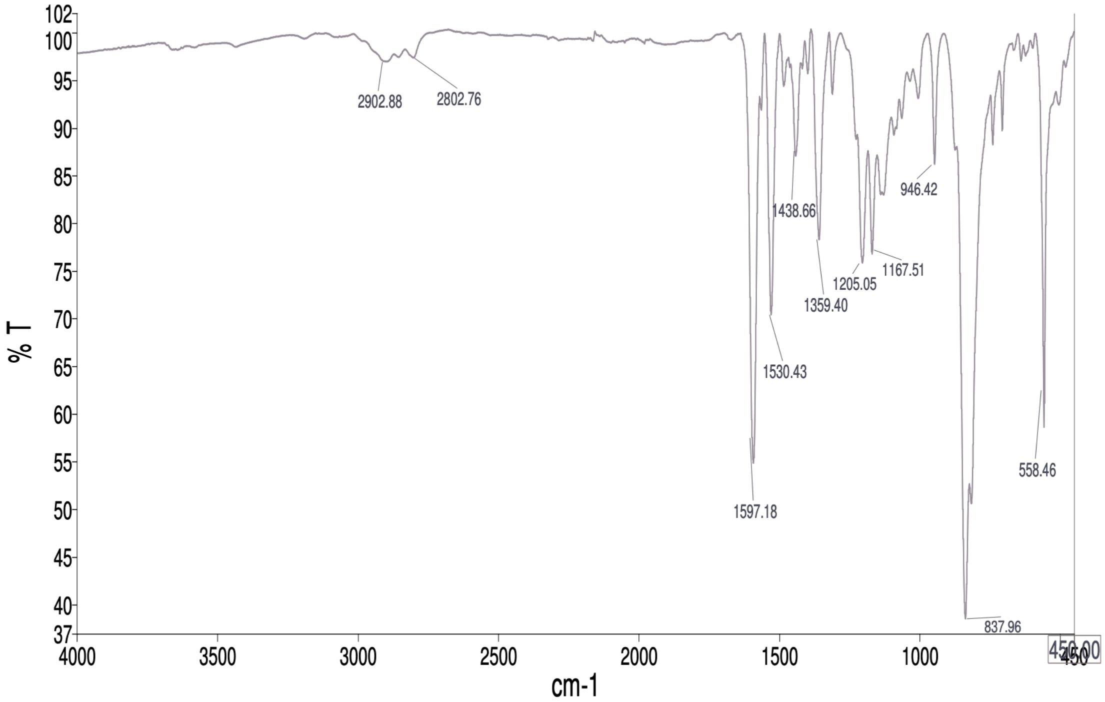

Figure S21. IR spectrum of solid [Cu(**1**)$_2$][PF$_6$].

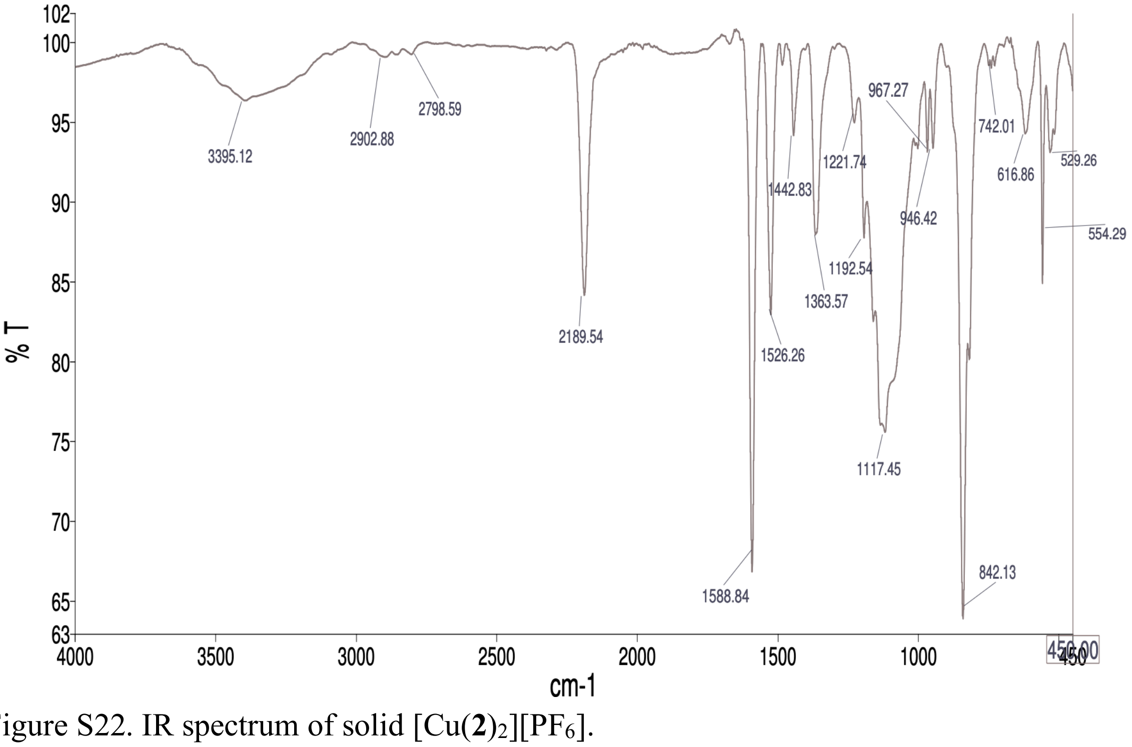

Figure S22. IR spectrum of solid [Cu(**2**)$_2$][PF$_6$].

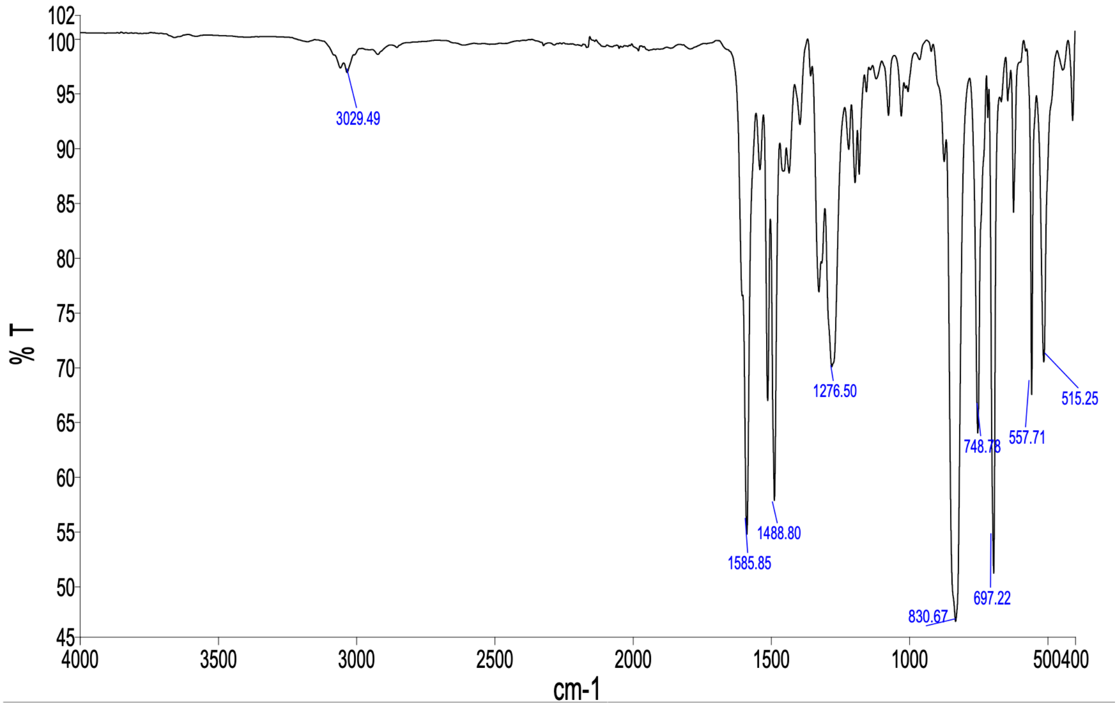

Figure S23. IR spectrum of solid $[Cu(\mathbf{3})_2][PF_6]$.

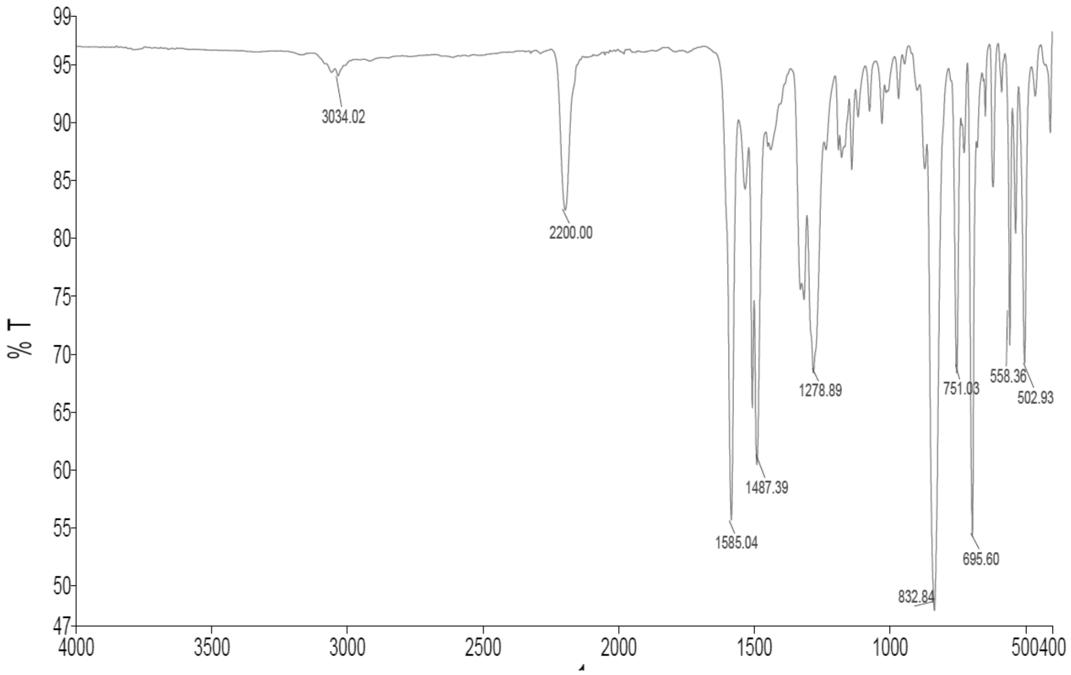

Figure S24. IR spectrum of solid $[Cu(\mathbf{4})_2][PF_6]$.

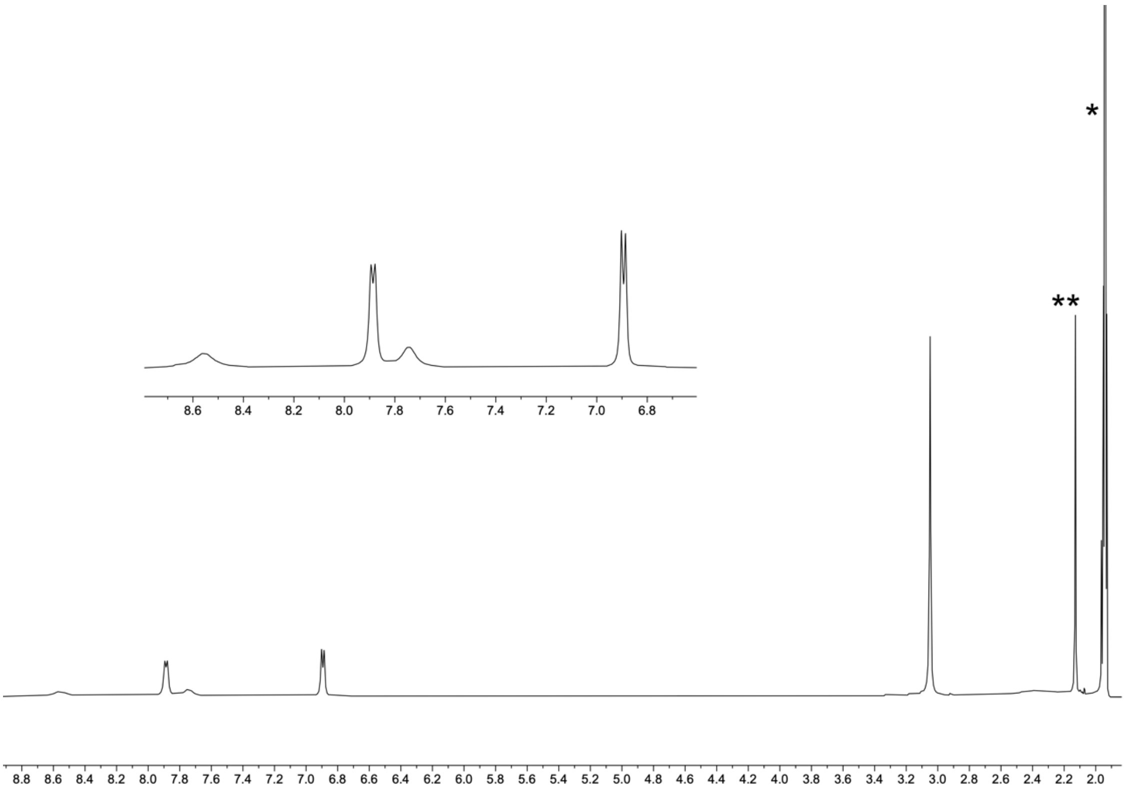

Figure S25. ^{1}H NMR spectrum (500 MHz, CD_3CN, 298 K) of [Cu(**1**)$_2$][PF_6] with inset of the aromatic region. * = residual CHD_2CN; ** = H_2O. Scale: δ / ppm

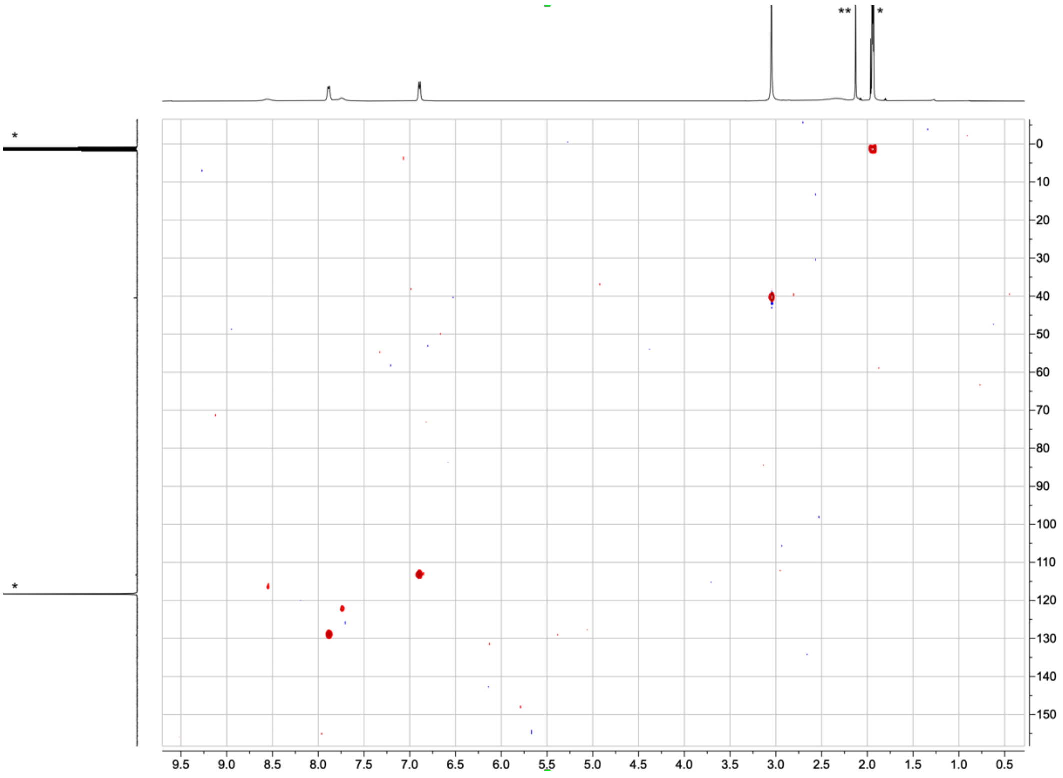

Figure S26. HMQC spectrum (500 MHz ^{1}H, 126 MHz ^{13}C, CD_3CN, 298 K) of [Cu(**1**)$_2$][PF_6]. * = CD_3CN or residual CHD_2CN; ** = H_2O. Scale: δ / ppm

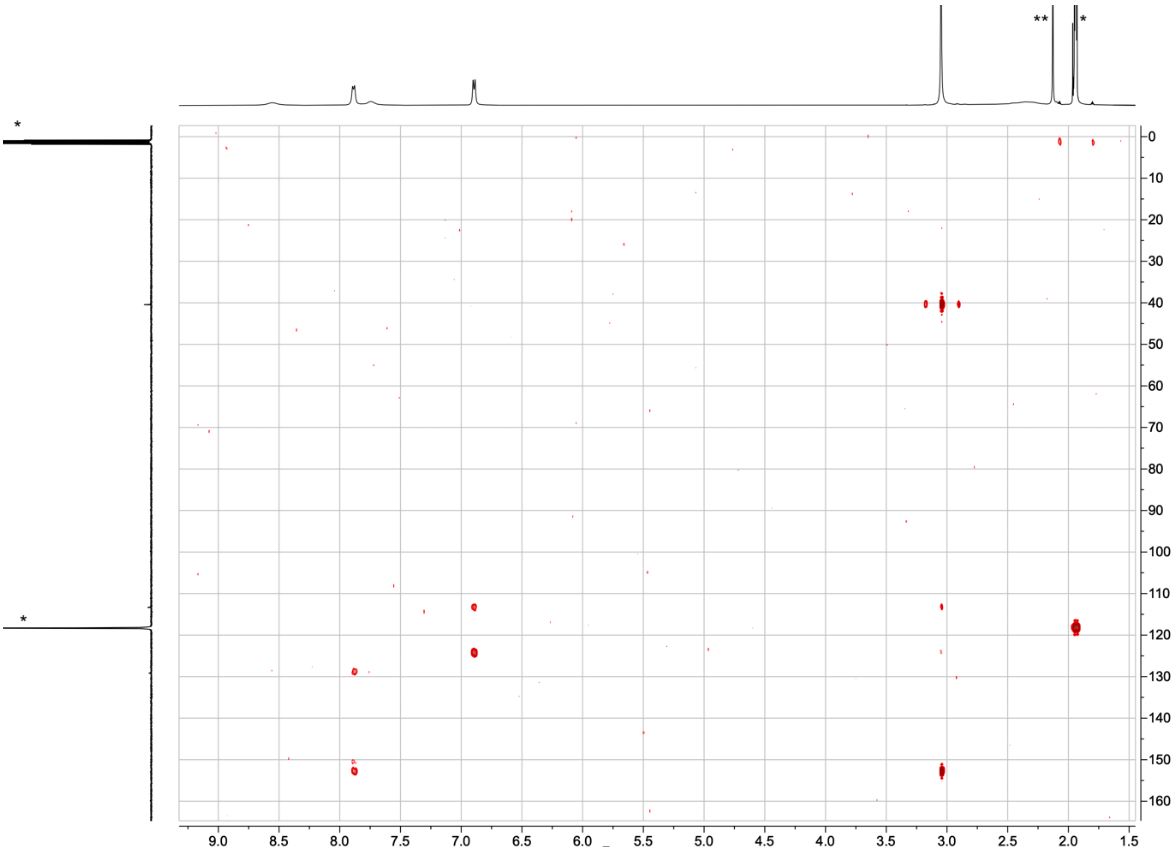

Figure S27. HMBC spectrum (500 MHz ^{1}H, 126 MHz ^{13}C, CD_3CN, 298 K) of [Cu(**1**)$_2$][PF_6]. * = CD_3CN or residual CHD_2CN; ** = H_2O. Scale: δ / ppm

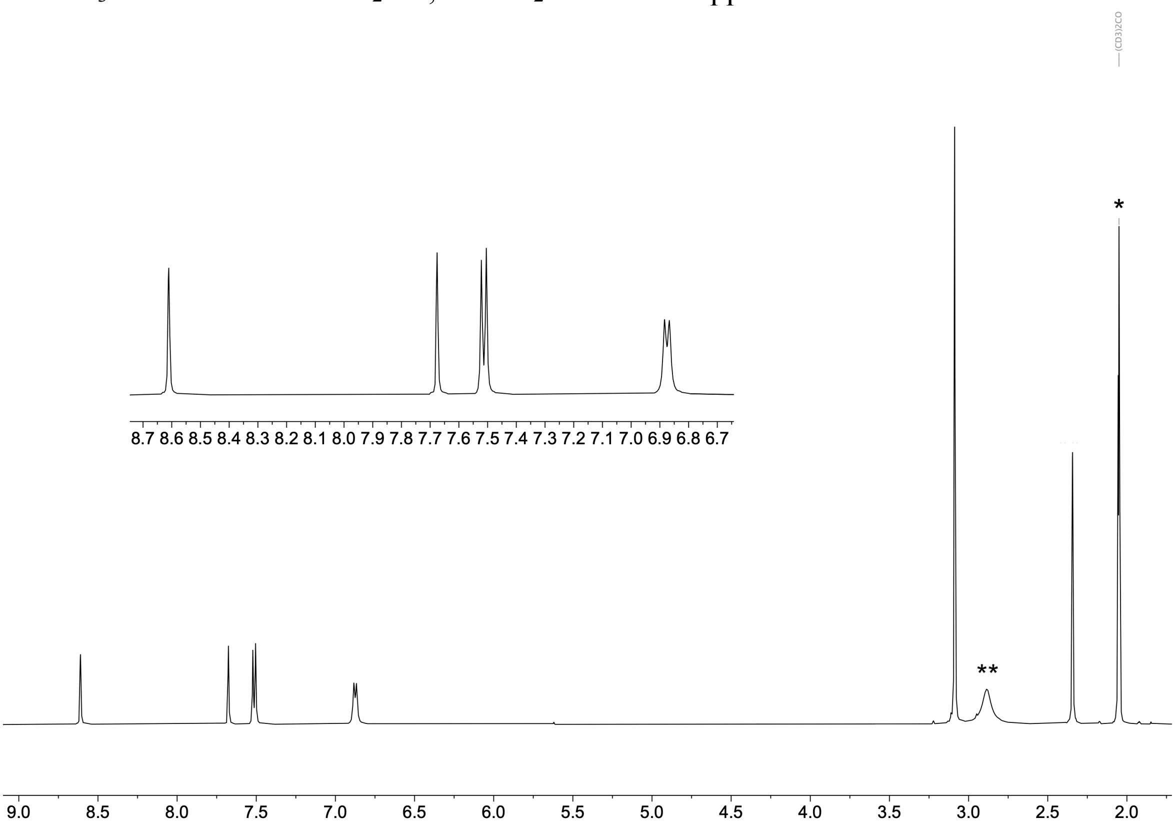

Figure S28. ^{1}H NMR spectrum (500 MHz, acetone-d_6, 298 K) of [Cu(**2**)$_2$][PF_6] with inset of the aromatic region. * = residual acetone-d_5; ** = H_2O. Scale: δ / ppm

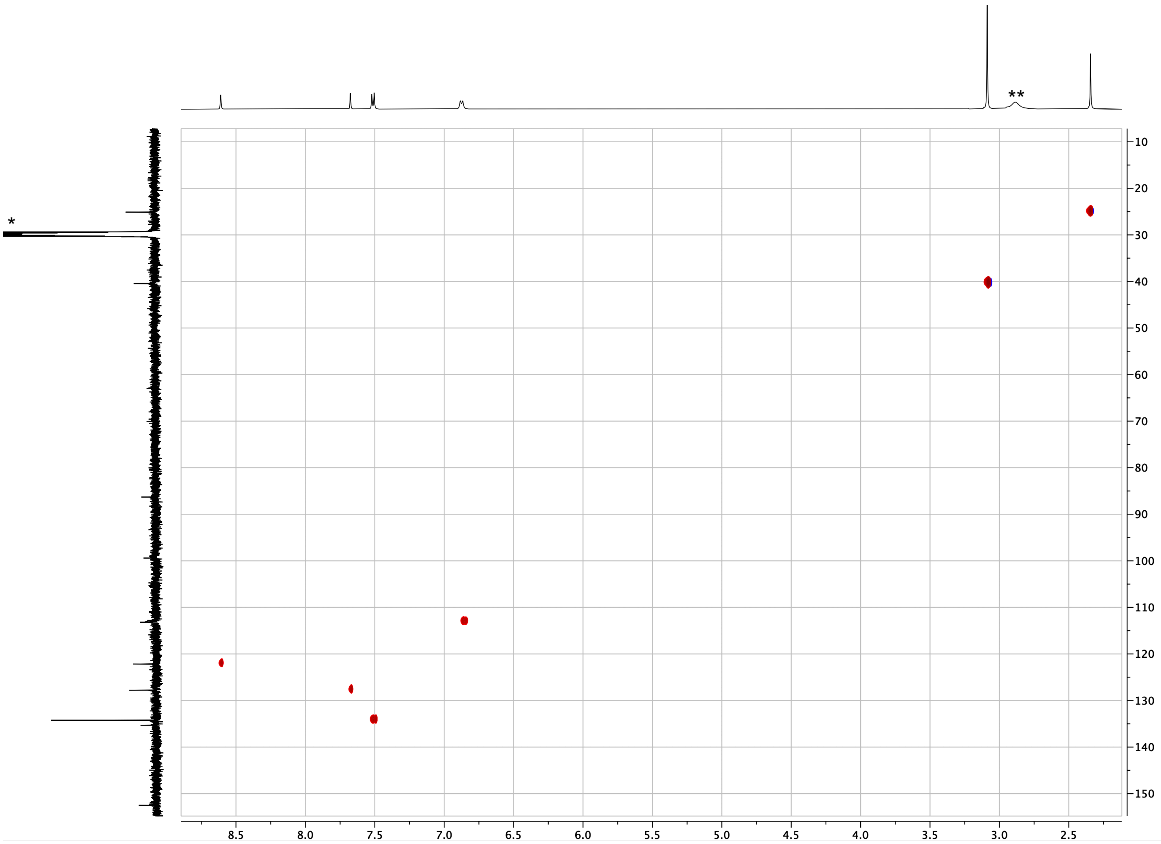

Figures S29. HMQC spectrum (500 MHz ^{1}H, 126 MHz ^{13}C, acetone-d_6, 298 K) of [Cu(**2**)$_2$][PF_6]. * = acetone-d_6; ** = H_2O. Scale: δ / ppm

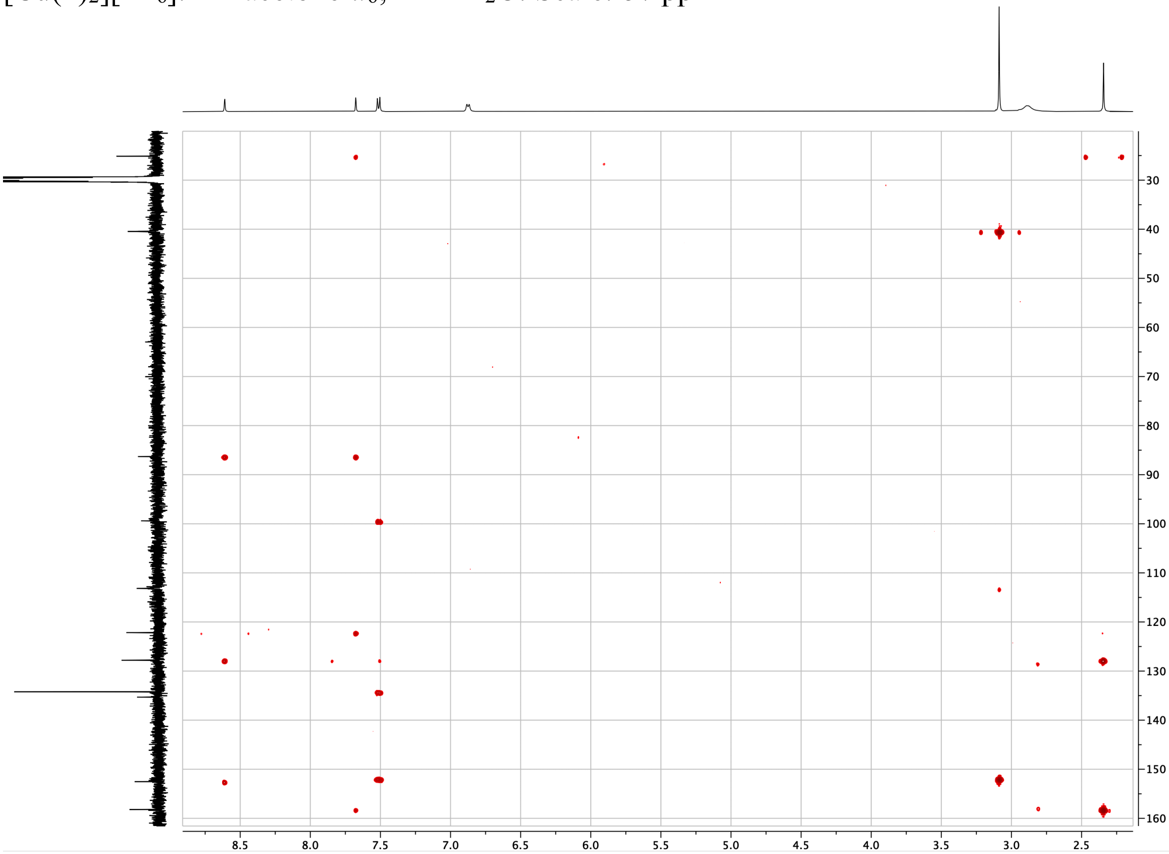

Figures S30. HMBC spectrum (500 MHz ^{1}H, 126 MHz ^{13}C, acetone-d_6, 298 K) of [Cu(**2**)$_2$][PF_6]. * acetone-d_6; ** = H_2O. Scale: δ / ppm

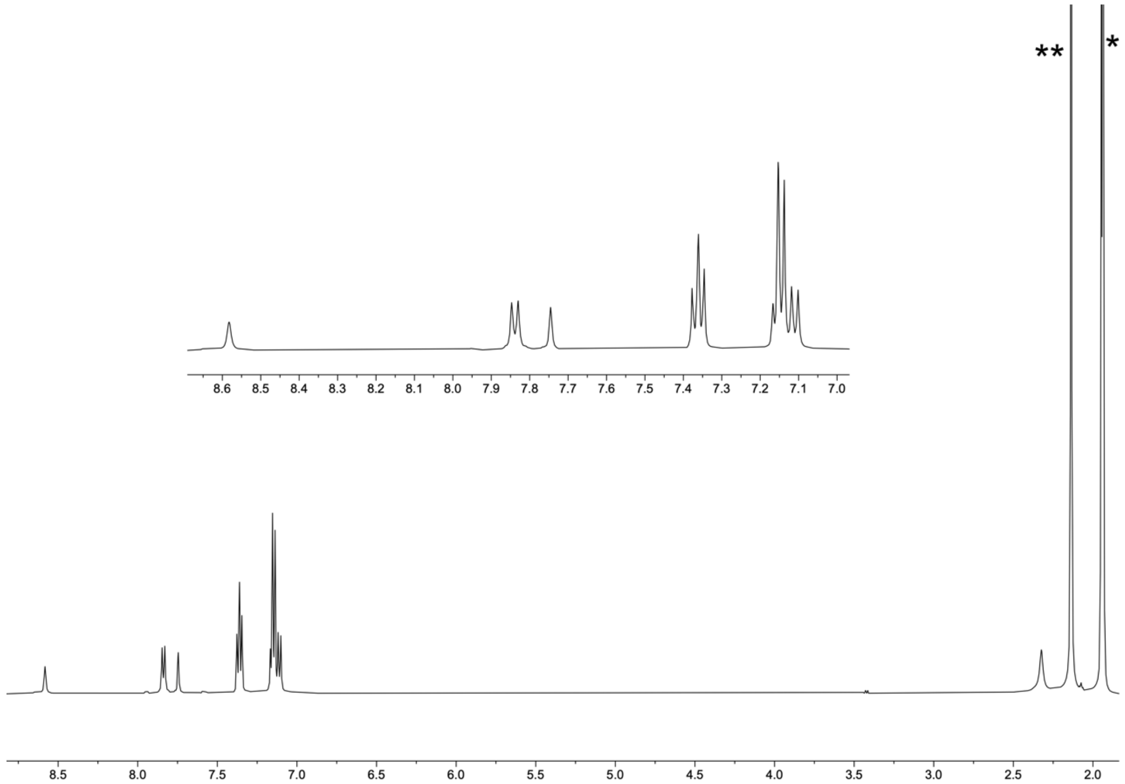

Figure S31. ^{1}H NMR spectrum (500 MHz, CD_3CN, 298 K) of [Cu(**3**)$_2$][PF_6] with inset of the aromatic region. * = residual CHD_2CN; ** = H_2O. Scale: δ / ppm

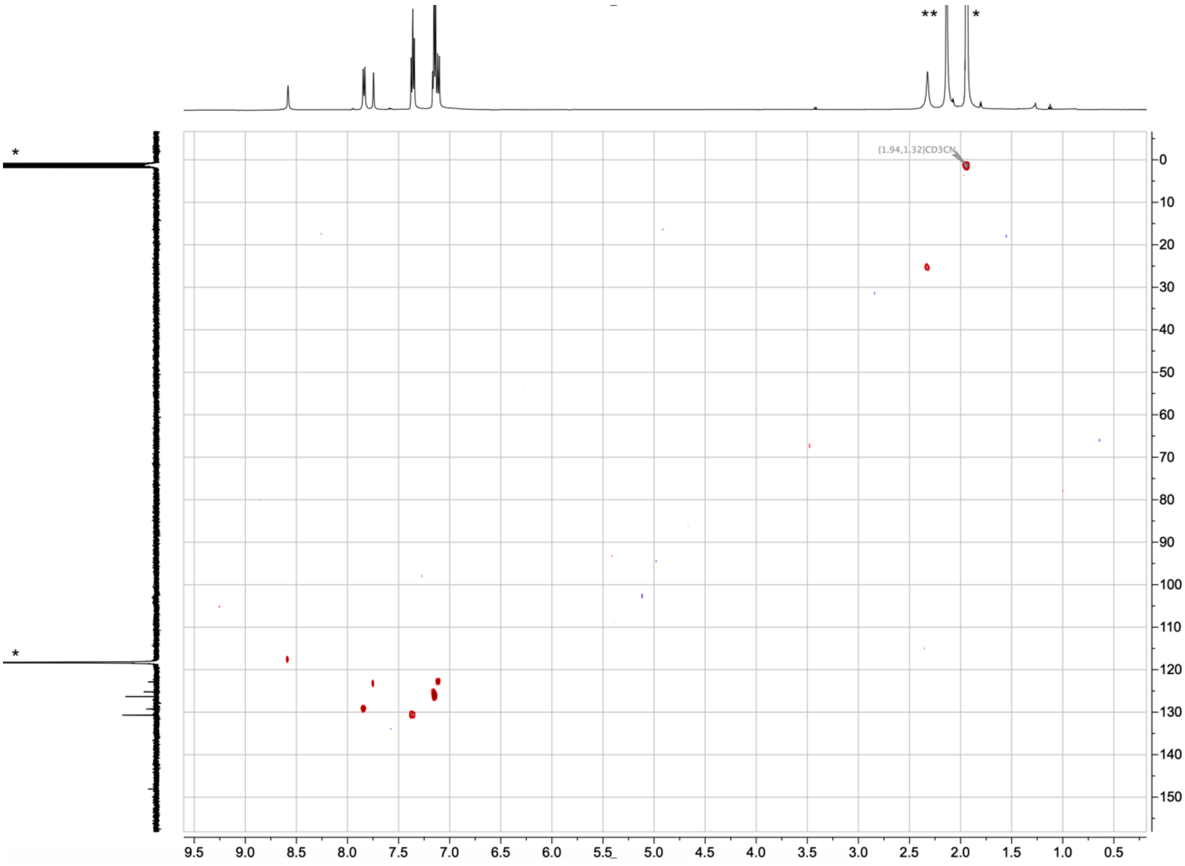

Figure S32. HMQC spectrum (500 MHz ^1H, 126 MHz ^{13}C, CD_3CN, 298 K) of $[Cu(\mathbf{3})_2][PF_6]$. * = CD_3CN or residual CHD_2CN; ** = H_2O. Scale: δ / ppm

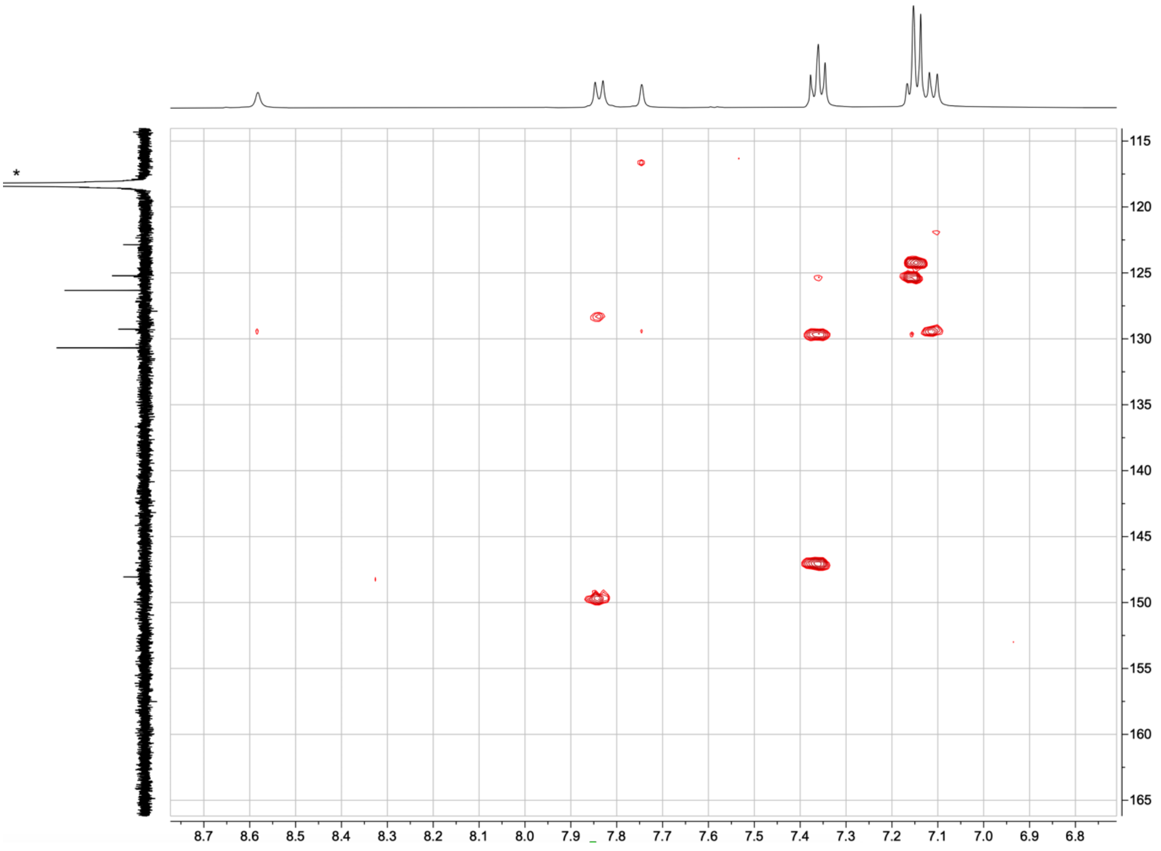

Figure S33. Part of the HMBC spectrum (500 MHz ^{1}H, 126 MHz ^{13}C, CD_3CN, 298 K) of [Cu(**3**)$_2$][PF_6]. * = CD_3CN or residual CHD_2CN. Scale: δ / ppm

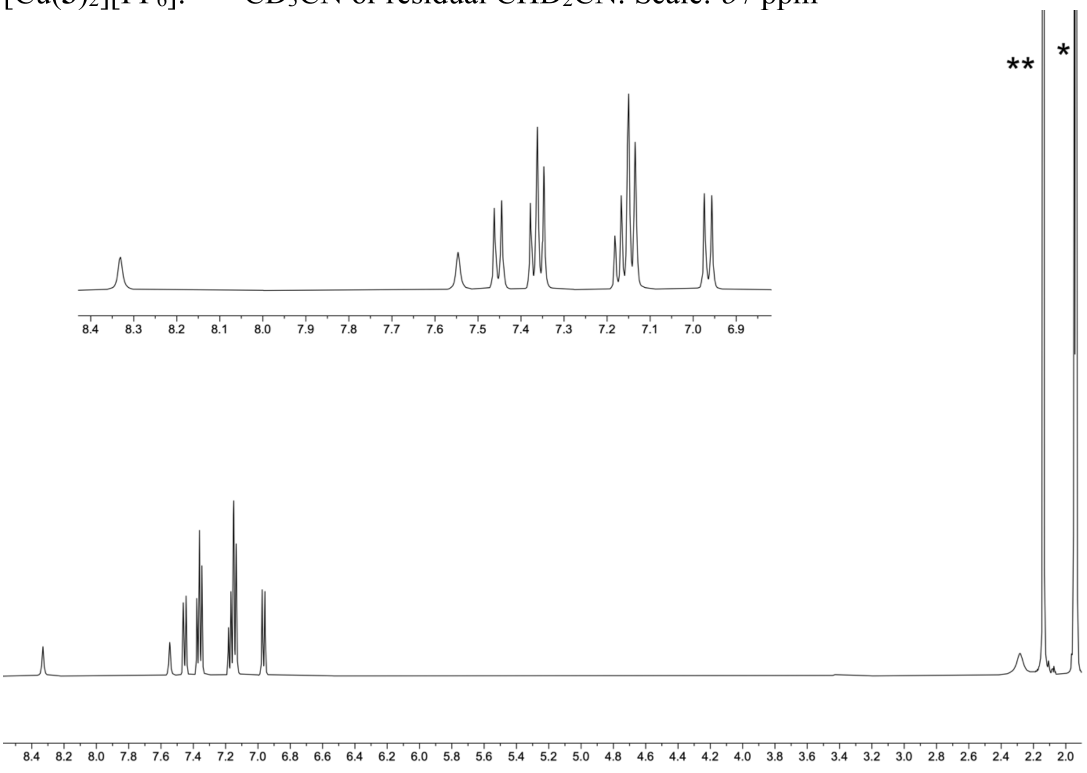

Figure S34. ^{1}H NMR spectrum (500 MHz, CD_3CN, 298 K) of [Cu(**4**)$_2$][PF_6] with inset of the aromatic region. * = residual CHD_2CN; ** = H_2O. Scale: δ / ppm

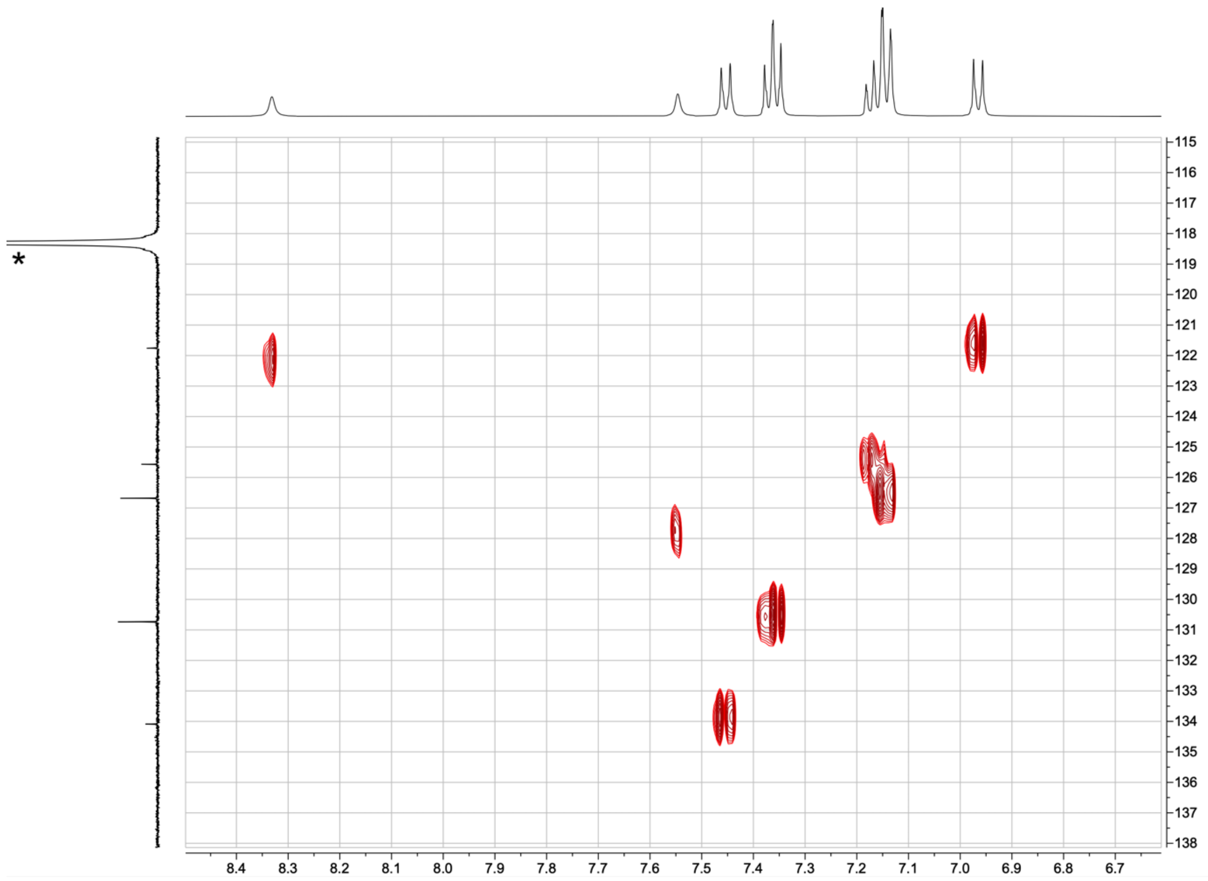

Figure S35. The aromatic region of the HMQC spectrum (500 MHz ^{1}H, 126 MHz ^{13}C, CD_3CN, 298 K) of $[Cu(\mathbf{4})_2][PF_6]$. * = CD_3CN. Scale: δ / ppm

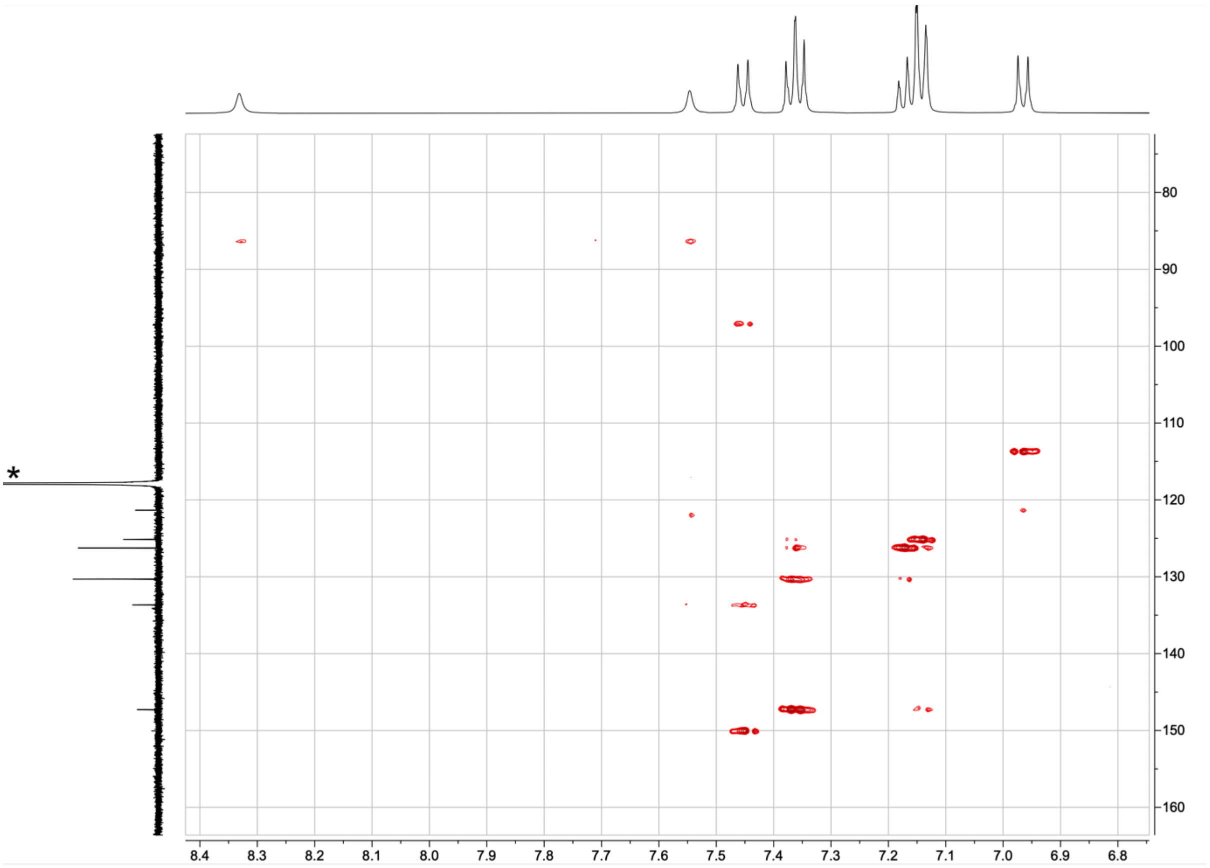

Figure S36. The aromatic region of the HMBC spectrum (500 MHz ^{1}H, 126 MHz ^{13}C, CD_3CN, 298 K) of $[Cu(\mathbf{4})_2][PF_6]$. * = CD_3CN. Scale: δ / ppm

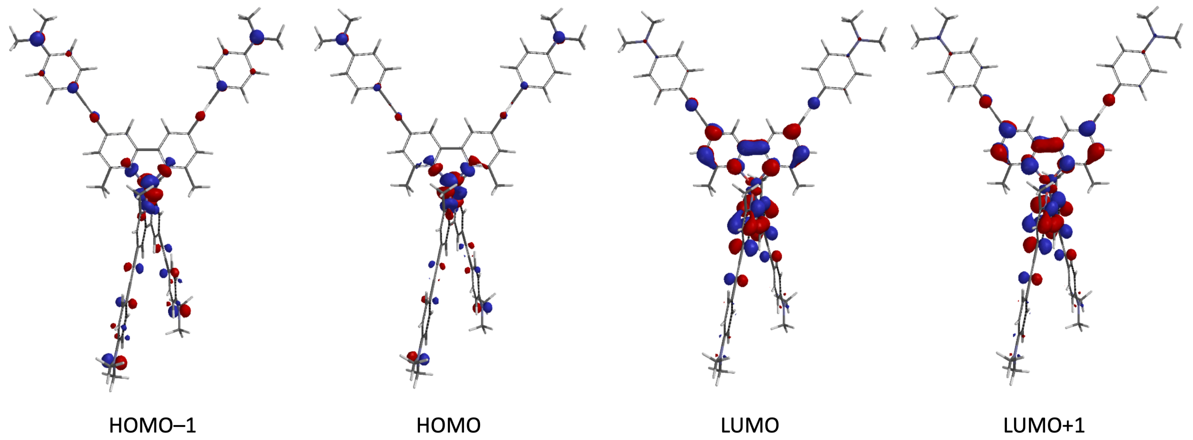

Figure S37. Orbital compositions of the highest occupied and lowest unoccupied molecular orbitals in $[Cu(\mathbf{2})_2]^{+}$ using a polarizable continuum solvation model ($CHCl_3$).

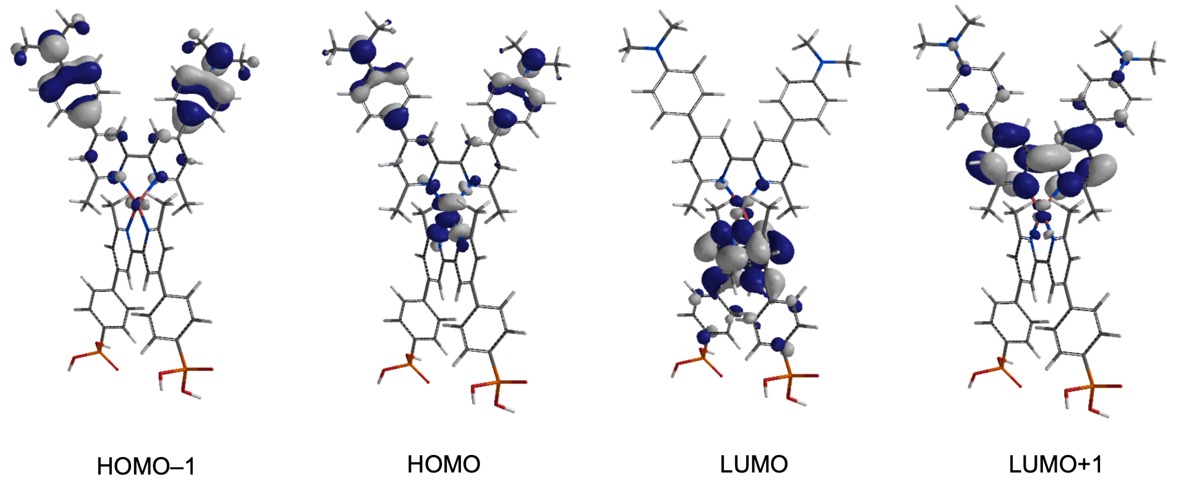

Figure S38. Orbital compositions of the highest occupied and lowest unoccupied molecular orbitals in [Cu(**5**)(**1**)]$^+$ calculated using DFT (B3LYP level, 6-31G* basis set, in vacuum). Geometry optimization was at the same level of calculation.

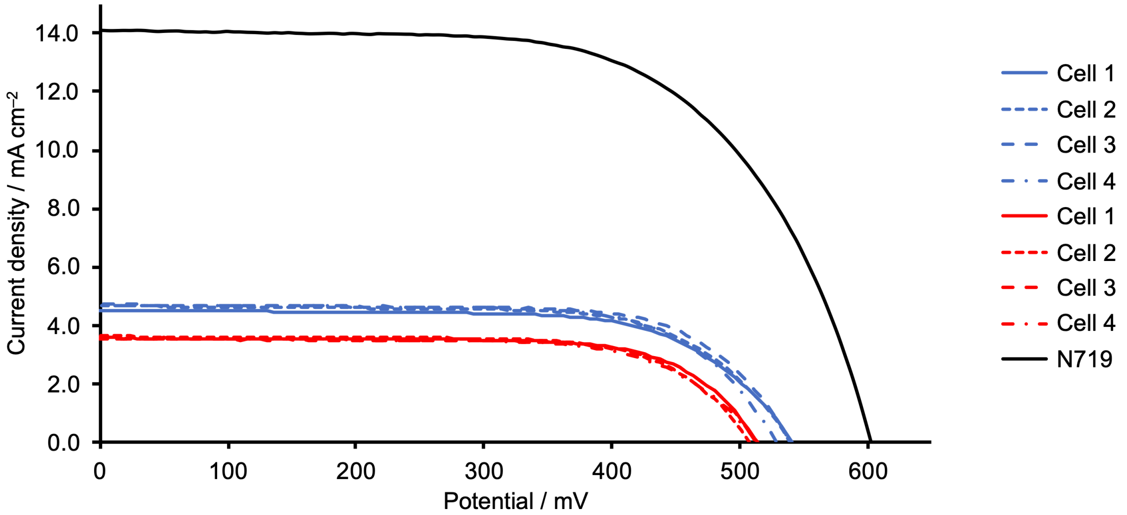

Figure S39. *J*–*V* curves for sets of four DSCs sensitized with [Cu(**5**)(**1**)]$^+$ (blue curves) and [Cu(**5**)(**2**)]$^+$ (red curves) measured on the day of sealing the cells and compared to the reference cell with N719.

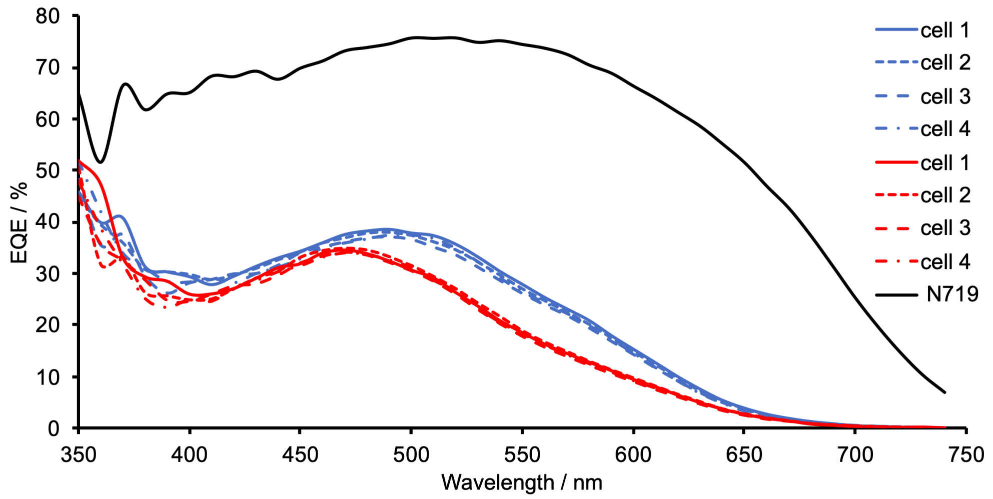

Figure S40. EQE spectra for sets of four DSCs sensitized with [Cu(**5**)(**1**)]$^+$ (blue curves) and [Cu(**5**)(**2**)]$^+$ (red curves) measured on the day of sealing the cells and compared to the reference cell with N719.

Table S1. EIS parameters obtained from fitting the experimental data for multiple DSCs, sets of four cells containing the dyes [Cu(**5**)(**1**)]$^+$ and [Cu(**5**)(**2**)]$^+$, and [Cu(**5**)(**1**)]$^+$ with the co-adsorbant *n*-decylphosphonic acid (DPA).

**Dye**	**Cell number**	**R_{rec} / Ω**	**C_μ / μF**	**R_{tr} / Ω**	**τ / ms**	**$τ_t$ / ms**	**Ld / μm**	**R_s / Ω**	**R_{Pt} / Ω**	**C_{Pt} / μF**	**J_{SC} / mA cm^{-2}**	**V_{OC} / mV**	***ff* / %**	***η* / %**
[Cu(**5**)(**1**)]$^+$	1	114	375	33	43	13	22	13	24	6	4.54	541	67.6	1.66
[Cu(**5**)(**1**)]$^+$	2	125	386	28	48	11	25	20	24	5	4.69	539	68.1	1.72
[Cu(**5**)(**1**)]$^+$	3	133	372	23	49	9	29	10	18	6	4.74	539	70.1	1.79
[Cu(**5**)(**1**)]$^{+\,a}$	4	120	391	19	47	7	30	10	18	5	4.72	528	69.0	1.72
[Cu(**5**)(**2**)]$^+$	1	181	317	39	57	12	26	9	19	6	3.59	514	70.7	1.30
[Cu(**5**)(**2**)]$^+$	2	192	315	37	60	12	27	9	20	6	3.64	508	69.6	1.29
[Cu(**5**)(**2**)]$^+$	3	200	328	30	66	10	31	10	16	6	3.54	513	71.2	1.29
[Cu(**5**)(**2**)]$^+$	4	159	284	44	45	12	23	11	24	6	3.57	514	68.4	1.25
[Cu(**5**)(**1**)]$^+$ + DPA	1	320	247	35	79	9	36	11	14	5	4.75	534	65.1	1.65
[Cu(**5**)(**1**)]$^+$ + DPA	2	384	252	39	97	10	38	10	21	6	4.75	548	68.9	1.79
[Cu(**5**)(**1**)]$^+$ + DPA	3	293	313	30	92	10	37	10	20	6	4.42	545	65.9	1.59
[Cu(**5**)(**1**)]$^+$ + DPA	4	382	259	53	99	14	32	10	29	5	4.73	543	68.8	1.77

[a]For the EIS measurements, a new cell 4 containing [Cu(**5**)(**1**)]$^+$ was prepared and values of J_{SC}, V_{OC}, *ff* and *η* do not correspond to the values in Table 2.
